# Transcriptomic and proteomic analyses of a new cytoplasmic male sterile line with a wild *Gossypium bickii* genetic background

**DOI:** 10.1186/s12864-020-07261-y

**Published:** 2020-12-02

**Authors:** Haiyan Zhao, Jianshe Wang, Yunfang Qu, Renhai Peng, Richard Odongo Magwanga, Fang Liu, Jinling Huang

**Affiliations:** 1 College of Agriculture, Shanxi Agricultural University, Taigu, 030801 Shanxi China; 2 School of Biotechnology and Food Engineering, Anyang Institute of Technology, Anyang, 455000 Henan China; 3 State Key Laboratory of Cotton Biology/Institute of Cotton Research, Chinese Academy of Agricultural Science, Anyang, 455000 Henan China

**Keywords:** Triple hybrids, Cytoplasmic male sterility, *Gossypium bickii*, Transcriptomic, Proteomics

## Abstract

**Background:**

Cotton is an important fiber crop but has serious heterosis effects, and cytoplasmic male sterility (CMS) is the major cause of heterosis in plants. However, to the best of our knowledge,

no studies have investigated CMS Yamian A in cotton with the genetic background of Australian wild *Gossypium bickii.* Conjoint transcriptomic and proteomic analysis was first performed between Yamian A and its maintainer Yamian B.

**Results:**

We detected 550 differentially expressed transcript-derived fragments (TDFs) and at least 1013 proteins in anthers at various developmental stages. Forty-two TDFs and 11 differentially expressed proteins (DEPs) were annotated by analysis in the genomic databases of *G. austral*, *G. arboreum* and *G. hirsutum*. Gene Ontology and Kyoto Encyclopedia of Genes and Genomes pathway analyses were performed to better understand the functions of these TDFs and DEPs. Transcriptomic and proteomic results showed that UDP-glucuronosyl/UDP-glucosyltransferase, 60S ribosomal protein L13a-4-like, and glutathione S-transferase were upregulated; while heat shock protein Hsp20, ATPase, F0 complex, and subunit D were downregulated at the microspore abortion stage of Yamian A. In addition, several TDFs from the transcriptome and several DEPs from the proteome were detected and confirmed by quantitative real-time PCR as being expressed in the buds of seven different periods of development. We established the databases of differentially expressed genes and proteins between Yamian A and its maintainer Yamian B in the anthers at various developmental stages and constructed an interaction network based on the databases for a comprehensive understanding of the mechanism underlying CMS with a wild cotton genetic background.

**Conclusion:**

We first analyzed the molecular mechanism of CMS Yamian A from the perspective of omics, thereby providing an experimental basis and theoretical foundation for future research attempting to analyze the abortion mechanism of new CMS with a wild *Gossypium bickii* background and to realize three-line matching.

**Supplementary Information:**

The online version contains supplementary material available at 10.1186/s12864-020-07261-y.

## Background

Cotton is an important cash crop with high-quality fiber, edible oil, and protein that is primarily used as animal feed [1]. Heterosis in cotton is quite apparent and has been widely used in yield quality, and resistance studies [2]. The adoption of the production of hybrid seeds is the most important among various means of utilizing cotton heterosis. At present, castration in the production of hybrid seeds often relies on hand emasculation and male-sterile lines produced by such means as chemically induced male sterility, genic male sterility, and cytoplasmic male sterility (CMS) [3]. Production practice shows that CMS is an effective method for heterosis utilization in crops and is widely used to produce hybrid seeds because it eliminates the need for artificial emasculation, saves manpower and material resources, enhances the purity of hybrid seeds and increases the output of crops [3, 4]. Around the world, there were CMS line studies on cotton in the 1960s, and in the following years, a number of germplasms have been developed, such as *G. arboreum* L, *G. harknessii* Brandegee, *G. trilobum* (DC.) Skov., *G. hirsutum*, and *G. barbadense* L. However, to the best of our knowledge, there is no report on CMS in cotton with the genetic background of Australian wild *Gossypium bickii*, which has been reported despite the considerable effects of heterosis in cotton germplasm development*.*

In recent years, advancement in molecular technology has enabled breeders and molecular researchers to identify various plant transcription factors and genes and explore protein expression at the transcriptome and proteome levels in such research efforts as CMS studies of Chinese cabbage [5], turnip [6], *Cucumis melo* L. [7], cotton [8, 9], rice [10], and *Brassica napus* L. [11]. Transcriptomic analysis in cotton (CMS-D8) revealed that reactive oxygen species (ROS) were released from mitochondria and served as important signal molecules in the nucleus, causing the formation of abnormal tapetum [8]. Proteome analyses in cotton indicated that the differentially expressed proteins (DEPs) mainly involved in pyruvate, carbohydrate and fatty acid metabolism had been identified between the male-sterile line 1355A and the male-fertile line 1355B [9]. Integrated analysis of the transcriptome and proteome can provide a complete picture with regard regard to the molecular mechanism of CMS, and this analysis has been employed in Chinese cabbage [12], *Brassica napus* [13], pepper [14], and *Citrus suavissima* [15] studies involving CMS. The conjoint analysis of the transcriptome and proteome in Shaan2A CMS and its maintainer line indicated that the sterility gene from the mitochondrion might suppress the expression of relevant transcription factor genes in the nucleus, affecting early anther development [13]. There have been relatively few studies of the conjoint analysis of transcriptomic and proteomic changes in CMS cotton to date.

Yamian A was identified by the cotton breeding group of Shanxi Agricultural University, as a new and stable cytoplasmic male sterile line derived from triple hybrids of *Gossypium bickii*, *Gossypium arboreum* and *Gossypium hirsutum* Linn [16]. The male sterility mechanism of Yamian A-CMS (YA-CMS) has not been elucidated. In the current study, conjoint transcriptomic, proteomic and early cytological, physiological and biochemical analyses were first performed between Yamian A and its maintainer Yamian B (YB) to elucidate the mechanism of YA-CMS. We attempted to identify differentially expressed genes and proteins at different development stages of anthers, discuss the relationship between these differentially expressed genes and proteins and male sterility in YA-CMS, and explore the possible effects on microspore abortion of YA-CMS. The results of this study may help to elucidate the molecular mechanism of YA-CMS and improve our understanding of male sterility in cotton.

## Results

### Transcriptome analysis

#### Expression type of the differentially expressed fragments

cDNA amplified fragment length polymorphism (cDNA-AFLP) analysis was used to perform transcriptome research between Yamian A and Yamian B with buds before, in the middle of, and after the microspore abortion stage. A total of 256 primer combinations were screened, and 134 of them produced 550 differentially expressed fragments. These differentially expressed fragments not only showed differences in quantity but also distinctions in quality (Fig. S1). Expression types of the differentially expressed fragments in the buds between Yamian A and Yamian B mainly included fifteen independent sets (Table S1); fragments detected at only one of the three stages in Yamian A or Yamian B (Type 1–6), especially the band number of type 2, were the most common among all types; fragments detected at any two of the three stages in Yamian A or Yamian B (Type 7–11), for example, the 12 fragments of type 7, were detected at the buds of before and middle microspore abortion stages in Yamian A; fragments detected at one or any two of the three stages in Yamian A and Yamian B (Type 12–15), for example, the 20 fragments of type 12, were detected at the buds of the middle microspore abortion stage in Yamian A and Yamian B.

#### Homology analysis of differentially expressed fragments

One hundred thirty-two transcript-derived fragments (TDFs) selected from 550 differentially expressed fragments were recycled, cloned, and sequenced, and 99 fragments ultimately produced readable sequences (Table S2). The sizes of the 99 fragments were concentrated between 19 to 500 bp. According to the max identity and e value, sequence alignment of these 99 TDFs in the *G. austral* (G_2_G_2_) [17], *G. arboreum* (A_2_A_2_) [18] and *G. hirsutum* (AADD)_1_ [19] genomic databases revealed that 34 showed homology to genes with known functions, whereas 57 did not show homology to other sequences, and 8 displayed identity with unknown proteins. Sequence analysis indicated that some different TDFs derived from different primer combinations were searched for the same homologous sequences, such as homologous sequences of T26 and T27, and both were UDP-glucuronosyl/UDP-glucosyltransferase (UGT) (Table 1).

**Table 1:** Homology analysis of TDF sequences on cDNA - AFLP

Name	Length (bp)	Accession	Description	Max Identity	E Value
T1	272	Gar05G45090	UDP-glucuronosyl/UDP-glucosyltransferase	100	3.00E-60
T2	226	KAA3484033.1	60S ribosomal protein L13a-4-like	90.7	1.00E-20
T12	379	Gohir.D01G184400.1.p	UDP-glucuronosyl/UDP-glucosyltransferase	98.1	2.00E-41
T13	331	Gohir.D05G008900.1.p	PREDICTED: autophagy-related protein 11-like	100	4.00E-68
T14	282	KAA3484560.1	Retrovirus-related Pol polyprotein from transposon TNT 1–94	46.15	2.00E-09
T18	367	Gar05G25840	Ccc1 family	56.25	3.00E-22
T25	354	KAA3464041.1	ATP-dependent RNA helicase DHX36 isoform X2	100	3.00E-73
T26	381	Gohir.D11G100700.1.p	UDP-glucuronosyl/UDP-glucosyltransferase	99.19	1.00E-84
T27	381	Gohir.D11G100700.1.p	UDP-glucuronosyl/UDP-glucosyltransferase	99.19	3.00E-84
T39	155	Gohir.D05G202700.1.p	PREDICTED: probable disease resistance protein At4g33300	100	2.00E-27
T41	286	Gohir.A03G112300.1.p	U box domain	100	3.00E-46
T42	286	Gohir.A03G112300.1.p	U box domain	100	3.00E-46
T45	152	KAA3485461.1	vacuole membrane protein KMS1-like	96.43	2.00E-11
T51	348	Gar11G01680	NAC domain; NAC domain superfamily	98.26	5.00E-74
T52	348	Gar11G01680	NAC domain; NAC domain superfamily	99.13	2.00E-75
T53	297	Gohir.D12G262100.1.p	PREDICTED: calcium-dependent protein kinase 26-like	100	4.00E-44
T54	297	Gohir.D12G262100.1.p	PREDICTED: calcium-dependent protein kinase 26-like	98.99	4.00E-43
T55	348	Gar11G01680	NAC domain, NAC domain superfamily	96.52	2.00E-73
T58	331	Gar06G11140	Protein of unknown function DUF1764, eukaryotic	100	5.00E-49
T59	331	Gar06G11140	Protein of unknown function DUF1764, eukaryotic	100	2.00E-45
T60	330	Gar06G11140	Protein of unknown function DUF1764, eukaryotic	98.95	6.00E-36
T61	328	Gar06G11140	Protein of unknown function DUF1764, eukaryotic	98.95	1.00E-46
T62	331	Gar06G11140	Protein of unknown function DUF1764, eukaryotic	97.89	1.00E-48
T63	90	Gar05G22100	Interferon-related developmental regulator	100	3.00E-13
T67	256	Gar10G07450	Myb-like domain	100	5.00E-38
T69	146	Gar03G28270	uncharacterized protein LOC108476290	100	8.00E-06
T70	146	Gar03G28270	uncharacterized protein LOC108476290	100	8.00E-06
T73	170	Gar08G17900	protein BPS1, chloroplastic	92.06	7.00E-33
T74	170	Gar08G04770	Plant peroxidase	98.21	6.00E-34
T75	168	KAA3487221.1	Transposon Tf2–9 polyprotein	82.5	2.00E-17
T76	168	KAA3487221.1	Transposon Tf2–9 polyprotein	82.5	2.00E-17
T77	354	KAA3464041.1	ATP-dependent RNA helicase DHX36 isoform X2	98.26	3.00E-71
T78	354	KAA3464041.1	ATP-dependent RNA helicase DHX36 isoform X2	99.13	2.00E-72
T85	92	Gar09G06330	Reverse transcriptase domain	89.66	1.00E-11
T89	65	Gohir.D06G107150.1.p	Uncharacterised protein family Ycf15	95	2.00E-08
T90	473	Gar08G03730	F-box domain	97.92	1.00E-23
T92	227	Gar11G34830	Golgi apparatus membrane protein TVP23-like	98.31	5.00E-35
T93	70	KAA3480627.1	Gag protease polyprotein-like protein	91.3	4.00E−10
T94	84	KAA3461301.1	Retrovirus-related Pol polyprotein from transposon 17.6	80	1.00E-09
T95	347	Gar10G30060	NAD(P)-binding domain	100	4.00E-44
T96	347	Gohir.D10G225100.1.p	PREDICTED: UDP-glucuronic acid decarboxylase 6-like	100	2.00E-41
T97	347	Gohir.D10G225100.1.p	PREDICTED: UDP-glucuronic acid decarboxylase 6-like	100	2.00E-41

#### Gene ontology (GO) analysis of TDFs

The *G. austral* (G_2_G_2_) [17], *G. arboreum* (A_2_A_2_) [18], and *G. hirsutum* (AADD)_1_ [19] genomic databases were used to assign GO IDs to the genes based on the sequence of 99 TDFs, and GO annotation was performed to retrieve molecular function, biological process, and cellular component terms according to their function.

**Fig. 1:**
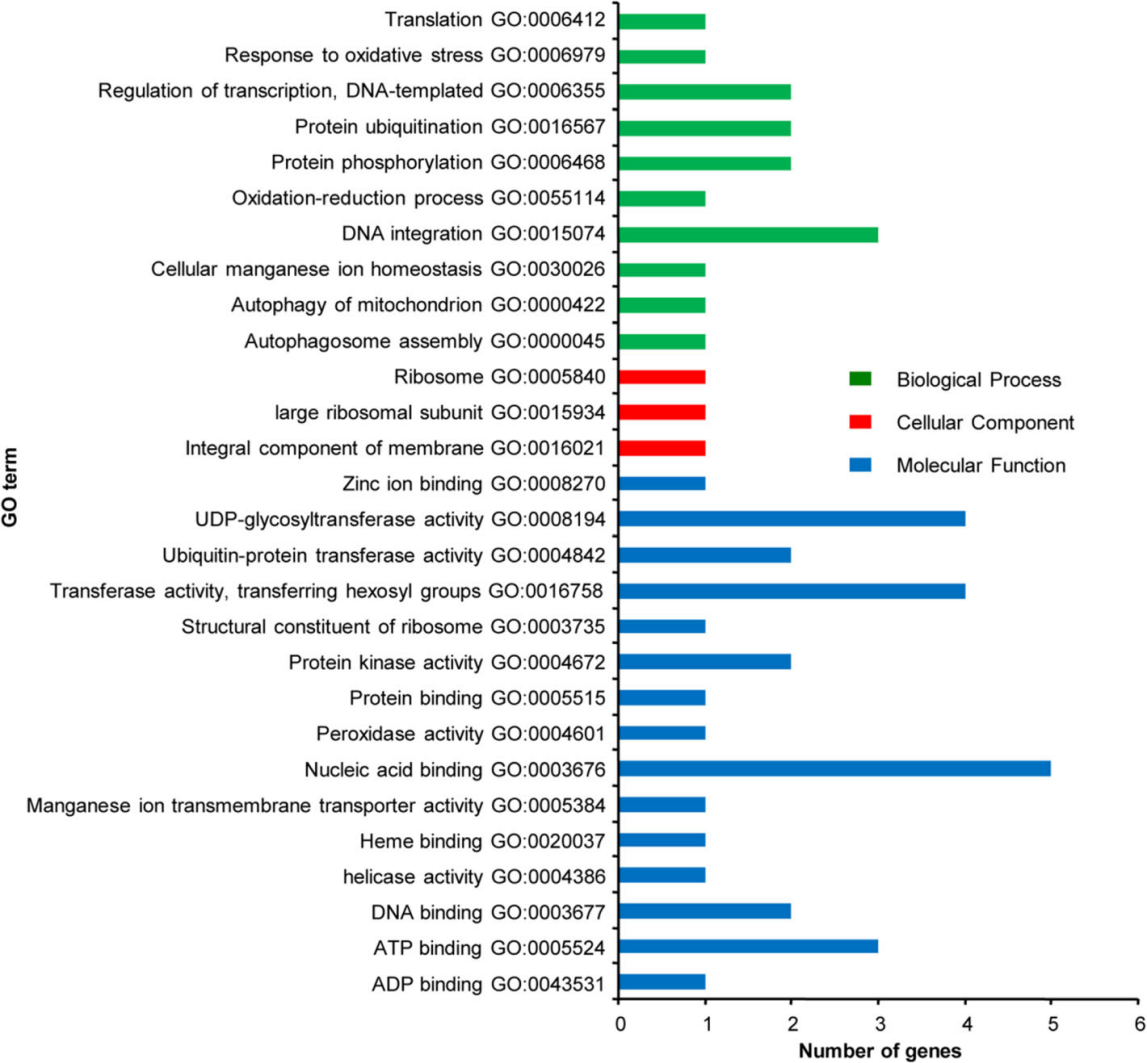
Gene Ontology analysis of TDFs on cDNA - AFLP

In terms of molecular function, these TDFs were assigned to 15 functional groups, in which the number of binding nucleic acids was 5; UDP-glycosyltransferase activity, transferase activity, and transferring hexosyl groups had 4; ATP binding had 3; ubiquitin-protein transferase activity, protein kinase activity, and DNA binding had 2; and zinc ion binding, structural constituent of ribosome, protein binding, peroxidase activity, manganese ion transmembrane transporter activity, heme binding, helicase activity, and ADP binding had 1 (Fig. 1).

In terms of biological processes, these TDFs were assigned to 10 functional groups, in which the number associated with DNA integration was 3; protein phosphorylation, protein ubiquitination, and regulation of transcription, DNA-templated had 2; and autophagy of mitochondrion, cellular manganese ion homeostasis, oxidation-reduction process, response to oxidative stress, translation, and autophagosome assembly had 1 (Fig. 1).

In terms of cellular components, these TDFs were assigned to 3 functional groups, in which the number of integral components of the membrane was 1, that of the large ribosomal subunit was 1, and that of the ribosome was 1 (Fig. 1).

#### Kyoto encyclopedia of genes and genomes (KEGG) pathway analysis of TDFs

Pathway assignment of TDFs was performed by the *G. austral* (G_2_G_2_) [17], *G. arboreum* (A_2_A_2_) [18] and *G. hirsutum* (AADD)_1_ [19] genomic databases. Ninety-nine TDFs were assigned to 7 KEGG pathways (Tab1e 2). The pathways with the most representation by TDFs were endocrine resistance (6), amino sugar and nucleotide sugar metabolism (2), ribosome (1), zeatin biosynthesis (1), autophagy - other (1), RNA degradation (1), and phenylpropanoid biosynthesis (1) (Table 2).

**Table 2:** KEGG pathway analysis of TDFs on cDNA – AFLP

Pathway (KO-ID)	Count	Name
Endocrine resistance (ko01522)	6	T2、T13、T18、T25、T26、T27
Ribosome (ko03010)	1	T2
Zeatin biosynthesis (ko00908)	1	T12
Autophagy - other (ko04136)	1	T13
RNA degradation (ko03018)	1	T25
Phenylpropanoid biosynthesis (ko00940)	1	T74
Amino sugar and nucleotide sugar metabolism (ko00520)	2	T95、T96

### Proteomics analysis

#### Protein expression profiles in Yamian a and Yamian B by 2-DE assay

Microspore abortion of YA-CMS occurred mainly between the stages of sporogenous cell and microsporocyte through the early-stage study of cell morphological observation and comparison of physiology and biochemistry characteristics [16]. According to bud development in cotton, the buds at the stages of sporogenous cell and microsporocyte in YA-CMS and YB were named A2, A3, B2, and B3 respectively. Thus, to further understand sterility mechanisms in YA-CMS, we performed a 2-DE analysis for the total protein of A2, A3, B2, and B3 (Fig. S2). The total concentration of all detected protein spots was determined via homogenization processing to obtain more accurate results. In total, 1013, 1110, 1112 and 1110 protein spots were detected in the 2-DE images of A2, B2, A3, and B3, respectively, by PDQuest8.0.1 software. The molecular weights of these proteins ranged from 10 to 100 kDa, and the isoelectric points ranged from 3.0 to 10.0.

**Fig. 2:**
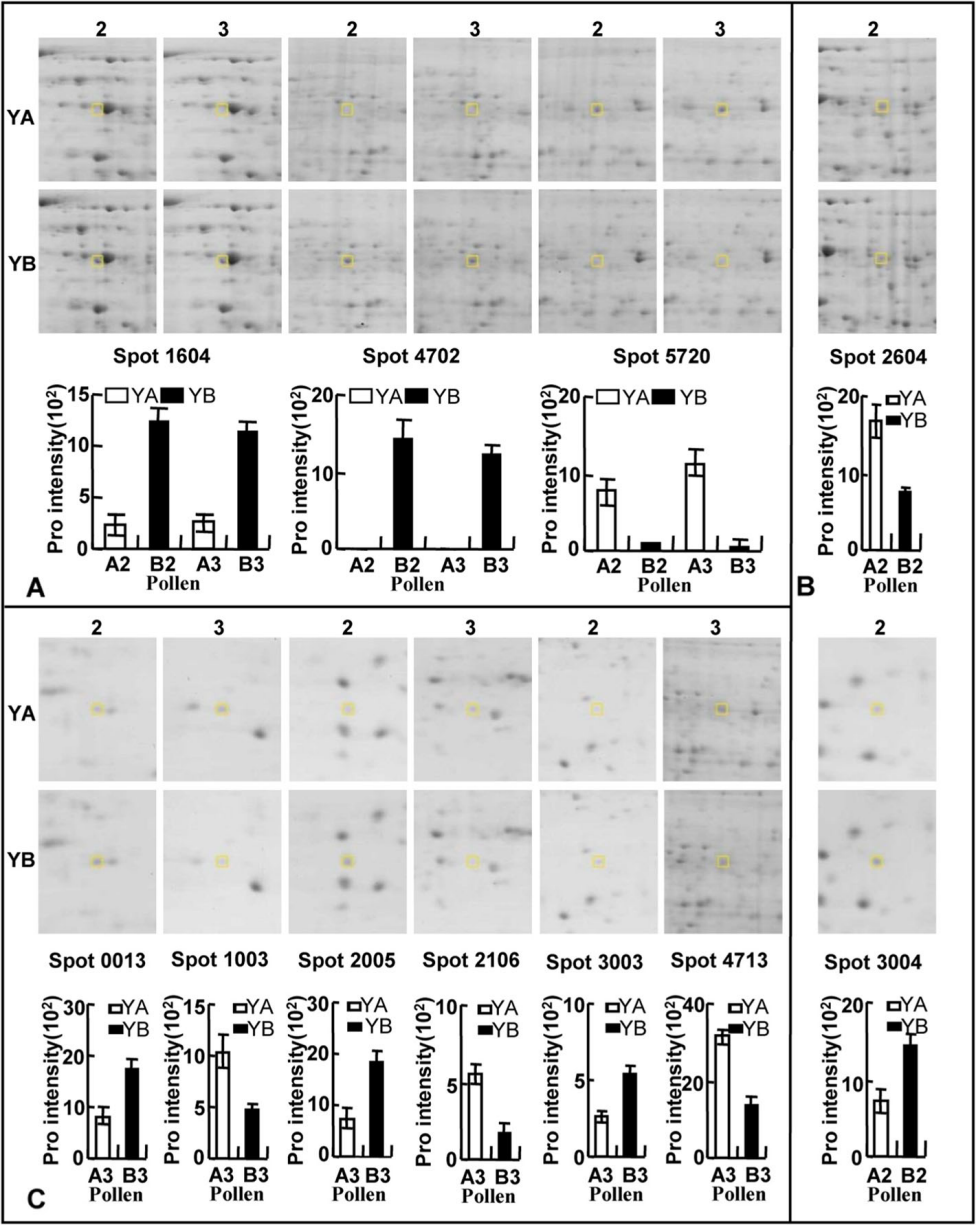
Enlarged differentially expressed protein spots from the pollen between YA-CMS and YB. A, B, C: represent differentially expressed proteins in sporogonium and microsporocyte stage, sporogonium stage, and microsporocyte stage from the pollen.

A total of 11 protein spots changed significantly (*P* < 0.05) in relative abundance by a minimum of a 2.0-fold change in at least one stage between YA-CMS and YB through point-to-point comparison and statistical analysis. Most of these differential spots displayed quantitative changes, but some displayed qualitative changes. Eight protein spots had significant quantitative differences in expression between YA-CMS and YB. For example, the 2604 spot was upregulated, whereas 3004 was downregulated in flower buds from the sporogenous cell stage of the YA-CMS plants, but instead in the YB plants. The 0013, 2005, and 3003 spots were upregulated whereas 1003, 2106, and 4713 were downregulated in flower buds from the microsporocyte stage of the YA-CMS plants but instead in the YB plants (Fig. 2). There were three protein spots that had significant qualitative differences in expression between YA-CMS and YB. For example, 1604 and 4702 were expressed only in flower buds from the sporogenous cell and microsporocyte stages of the YB plants but not in YA-CMS plants. The 5720 spot was detected only in flower buds from the sporogenous cell and the microsporocyte stages of the YA-CMS plants but not in YB plants (Fig. 2).

#### Identification and functional annotation of differentially expressed proteins (DEPs)

All 11 differentially expressed spots were analyzed by LC-Chip-ESI-QTOF-MS. Eleven spots were successfully identified by MASCOT and PEAKS 6.0 software searches of the *G. austral* (G_2_G_2_) [17], *G. arboreum* (A_2_A_2_) [18], *G. hirsutum* (AADD)_1_ [19] and *G. raimondii* (D_5_D_5_) [20] genomic databases according to the -10lgP and peptides. The 11 spots included ATPase, F0 complex, subunit D, mitochondrial (spot 0013), pathogenesis-related protein STH-2 (spot 1003), ATPase, F1/V1/A1 complex, alpha/beta subunit, nucleotide-binding domain (spots 1604, 4713), heat shock protein Hsp20 (spot 2005), glutathione S-transferase (spot 2106), enolase (spot 2604), peroxisomal membrane protein PMP22 (spot 3003), major latex protein domain (spot 3004), and ribulose bisphosphate carboxylase, large subunit (spots 4702, 5720) (Table 3).

**Table 3:** Identification of DEPs by MS/MS

SSP Numbers	Protein name	-10 lgP	Molecular (kDa)/pI	Sequence coverage (%)	Peptides	Accession no.
0013	ATPase, F0 complex, subunit D, mitochondrial	175.07	19.588/4.94	35	6	Cotton_D_gene_10017606
1003	Pathogenesis-related protein STH-2	198.11	17.154/5.16	52	6	Cotton_D_gene_10010317
1604	ATPase, F1/V1/A1 complex, alpha/beta subunit, nucleotide-binding domain	485.76	46.501/5.96	49	17	Gohir.1Z049799.1
2005	Heat shock protein Hsp20	127.41	17.514/6.04	21	4	Cotton_D_gene_10037266
2106	Glutathione S-transferase	65.2	25.847/5.84	11	2	Gohir.D09G157000.1
2604	Enolase	235.44	47.665/5.64	32	12	Cotton_D_gene_10005023
3003	Peroxisomal membrane protein PMP22	26.11	21.485/10.35	3	1	Cotton_D_gene_10021212
3004	Major latex protein domain	75.51	17.993/6.09	28	6	Cotton_D_gene_10014373
4702	Ribulose bisphosphate carboxylase, large subunit	178.73	53.894/6.57	25	12	Cotton_D_gene_10018308
4713	ATPase, F1/V1/A1 complex, alpha/beta subunit, nucleotide-binding domain	120.18	51.587/8.05	6	3	Cotton_D_gene_10036457
5720	Ribulose bisphosphate carboxylase, large subunit	191.28	53.894/6.57	31	15	Cotton_D_gene_10018308

**Fig. 3:**
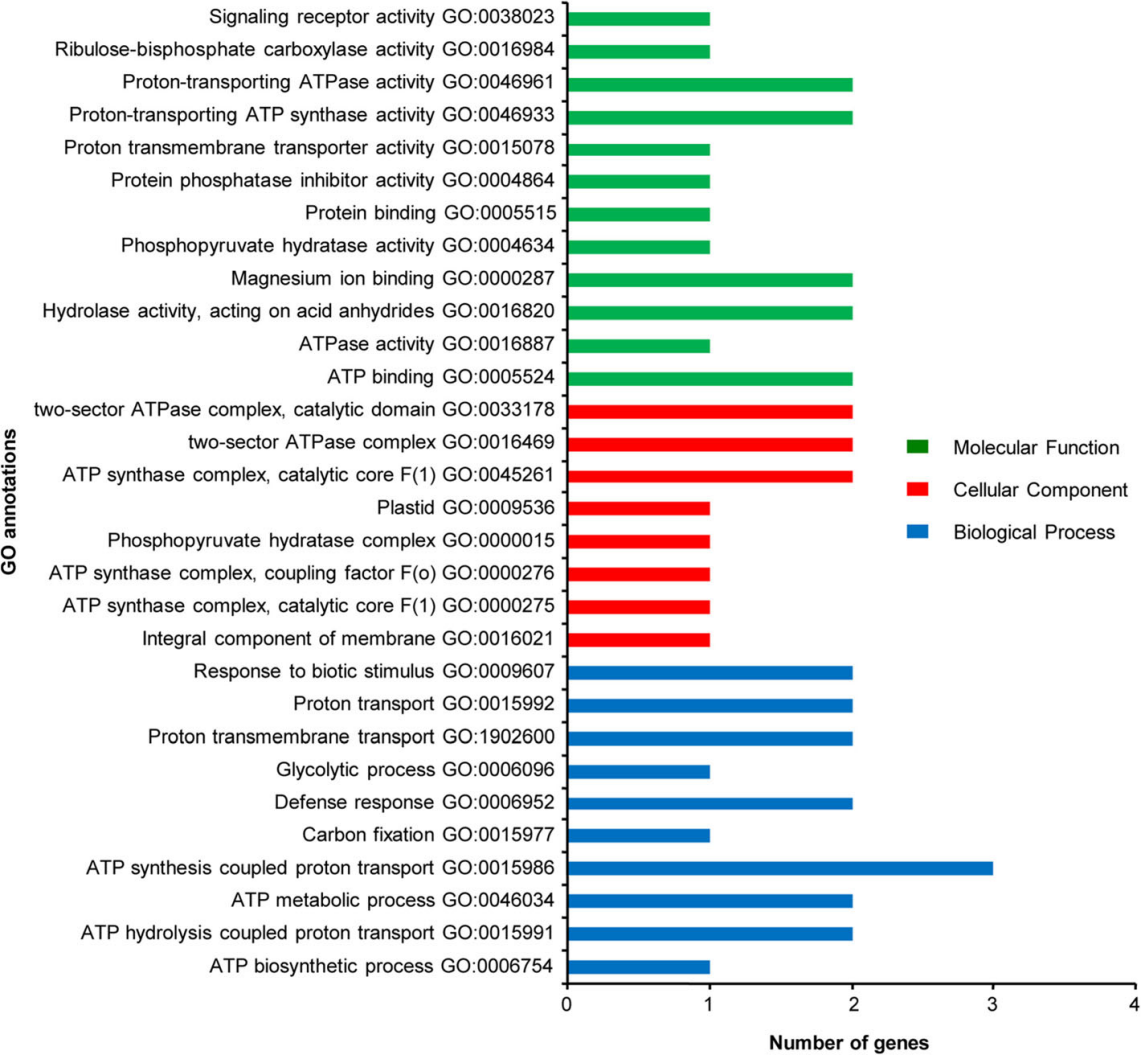
Gene Ontology analysis of differentially expressed proteins

GO annotations were performed to retrieve molecular function, biological process, and cellular component terms according to their function. *G. raimondii* (D_5_D_5_), *G. austral* (G_2_G_2_), *G. arboreum* (A_2_A_2_) and *G. hirsutum* (AADD)_1_ genomic databases were used to assign GO IDs to the 11 DEPs (Fig. 3).

In terms of molecular function, these DEPs were assigned to 12 functional groups, in which the numbers of ATP binding, hydrolase activity, acting on acid anhydrides, catalyzing transmembrane movement of substances, magnesium ion binding, proton-transporting ATP synthase activity, rotational mechanism, and proton-transporting ATPase activity, rotational mechanism DEPs were 2, and ATPase activity, phosphopyruvate hydratase activity, protein binding, protein phosphatase inhibitor activity, proton transmembrane transporter activity, ribulose-bisphosphate carboxylase activity, and signaling receptor activity were 1 (Fig. 3).

In terms of biological process, these DEPs were assigned to 10 functional groups, in which the number of ATP synthesis coupled proton transport DEPs was 3, ATP hydrolysis coupled proton transport, ATP metabolic process, defense response, proton transmembrane transport, proton transport, and response to biotic stimulus were 2, and ATP biosynthetic process, carbon fixation, and glycolytic process were 1(Fig. 3).

In terms of cellular components, these DEPs were assigned to 8 functional groups, in which the numbers of proton-transporting ATP synthase complex, catalytic core F (1), proton-transporting two-sector ATPase complex, and proton-transporting two-sector ATPase complex, catalytic domain DEPs were 2, and integral component of membrane, mitochondrial proton-transporting ATP synthase complex, catalytic core F (1), mitochondrial proton-transporting ATP synthase complex, coupling factor F(o), phosphopyruvate hydratase complex, and plastid were 1(Fig. 3).

The differential protein functions in the metabolic pathway were revealed by *G. raimondii* (D_5_D_5_), *G. austral* (G_2_G_2_), *G. arboreum* (A_2_A_2_) and *G. hirsutum* (AADD)_1_ genomic databases analysis. Eleven DEPs were assigned to 7 KEGG pathways (Table 4). The pathways with the most representation by DEPs were Oxidative phosphorylation (3), Carbon fixation in photosynthetic organisms (2), Glyoxylate and dicarboxylate metabolism (2), Glycolysis/Gluconeogenesis (1), Glutathione metabolism (1), Peroxisome (1), and Photosynthesis (1).

**Table 4:** KEGG pathway of differentially expressed proteins

Pathway (KO-ID)	Count	Protein
Glycolysis / Gluconeogenesis (ko00010)	1	2604
Glutathione metabolism (ko00480)	1	2106
Glyoxylate and dicarboxylate metabolism (ko00630)	2	4702、5720
Carbon fixation in photosynthetic organisms (ko00710)	2	4702、5720
Peroxisome (ko04146)	1	3003
Oxidative phosphorylation (ko00190)	3	4713、0013、1604
Photosynthesis (ko00195)	1	1604

**Fig. 4:**
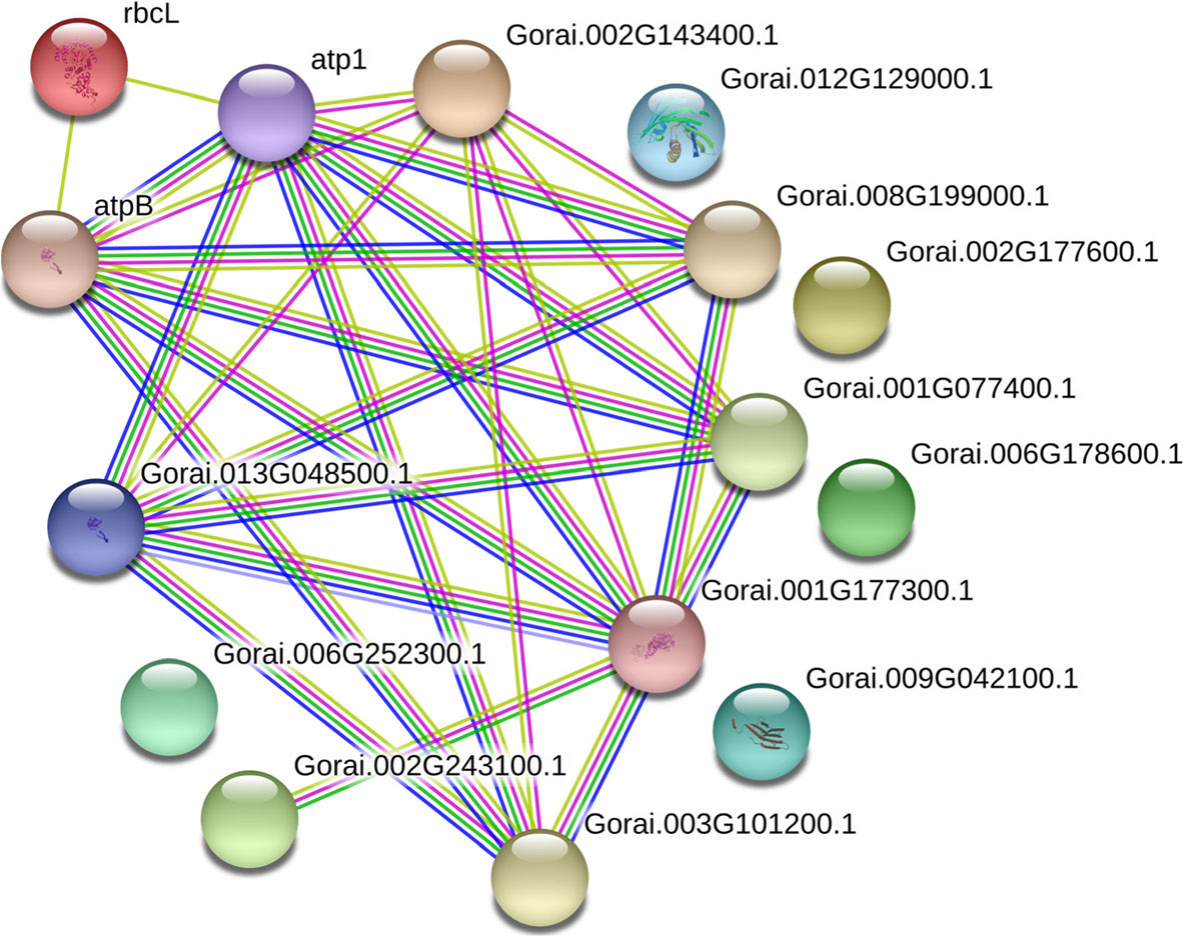
Biological interaction network of the differentially expressed proteins. Blue indicates co-occurrence evidence, red indicates fusion evidence, green indicates neighborhood evidence, yellow indicates text-mining evidence, purple indicates experimental evidence, light blue indicates database evidence and black indicates coexpression evidence

Insights into the functional mechanisms of living cells can be provided by protein-protein interaction networks. Selecting *G. raimondii* (D_5_D_5_) as the search species, 11 proteins were recognized as key nodes with various relationships in biological interaction networks by using the online tools of STRING 11.0 (http://string-db.org/cgi/input.pl). The results showed that there were interactions among atp1 (spot 4713), Gorai.002G143400.1 (spot 0013), Gorai.002G243100.1 (spot 2604), Gorai.013G048500.1 (spot 1604) and rbcL (spot 4702/5720), and there were no interactions among Gorai.012G129000.1 (spot 1003), Gorai.002G177600.1 (spot 3004), Gorai.006G178600.1 (spot 2106), Gorai.009G042100.1 (spot 2005), and Gorai.006G252300.1 (spot 3003) (Fig. 4).

#### Validation of genes and proteins of differential abundance

**Fig. 5:**
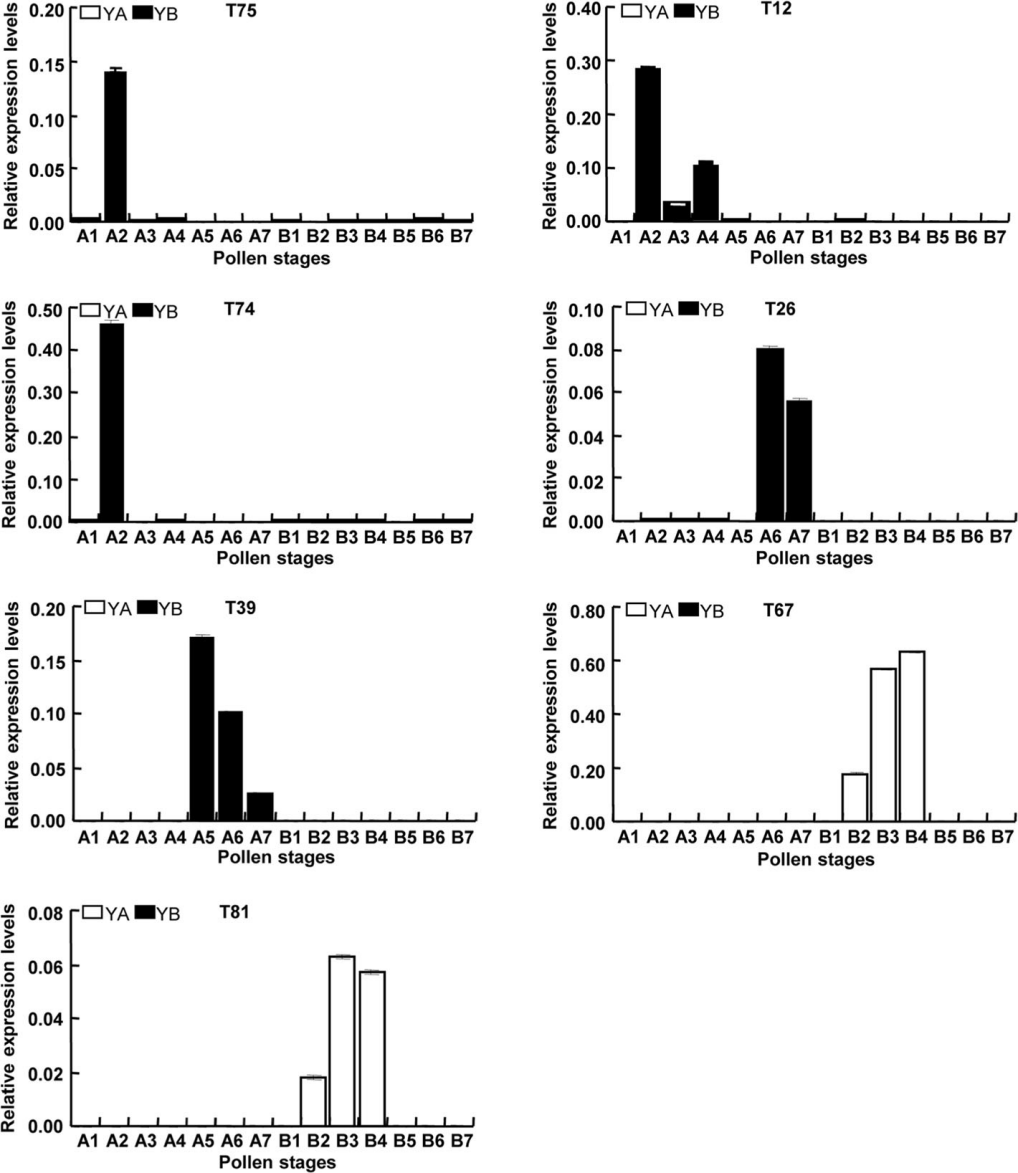
Validation of TDF sequences on cDNA-AFLP by qRT-PCR. A1-A7 and B1-B7 represent seven different development periods of the pollen on Yamian A and Yamian B, respectively

To verify the differential abundance of gene expression derived from cDNA-AFLP, seven genes were selected to perform quantitative real-time PCR (qRT-PCR) using equal amounts of cDNA templates from the buds of seven different development periods of both Yamian A and Yamian B. The results of qRT-PCR were the same as those obtained with cDNA-AFLP (Fig. S3 and Fig. 5). T75 and T74 were both detected at the 2nd stage of flower buds in Yamian A. T12 was detected at the 2nd, 3rd and 4th stage of flower buds in Yamian A, which was the peak period of microspore abortion, and it was not detected in the flower buds of other periods in Yamian A and all periods in Yamian B. T26 and T39 were detected at the 6th and 7th and at the 5th, 6th, and 7th stages of flower buds in Yamian A, respectively, which followed microspore abortion, and they were not detected in the flower buds of other periods in Yamian A and all periods in Yamian B. T67 and T81 were detected at the 2nd, 3rd and 4th stages of flower buds in Yamian B and were not detected in the flower buds of other periods in Yamian B and all periods in Yamian A. The qRT-PCR results of these selected genes were consistent with their cDNA-AFLP results.

**Fig. 6:**
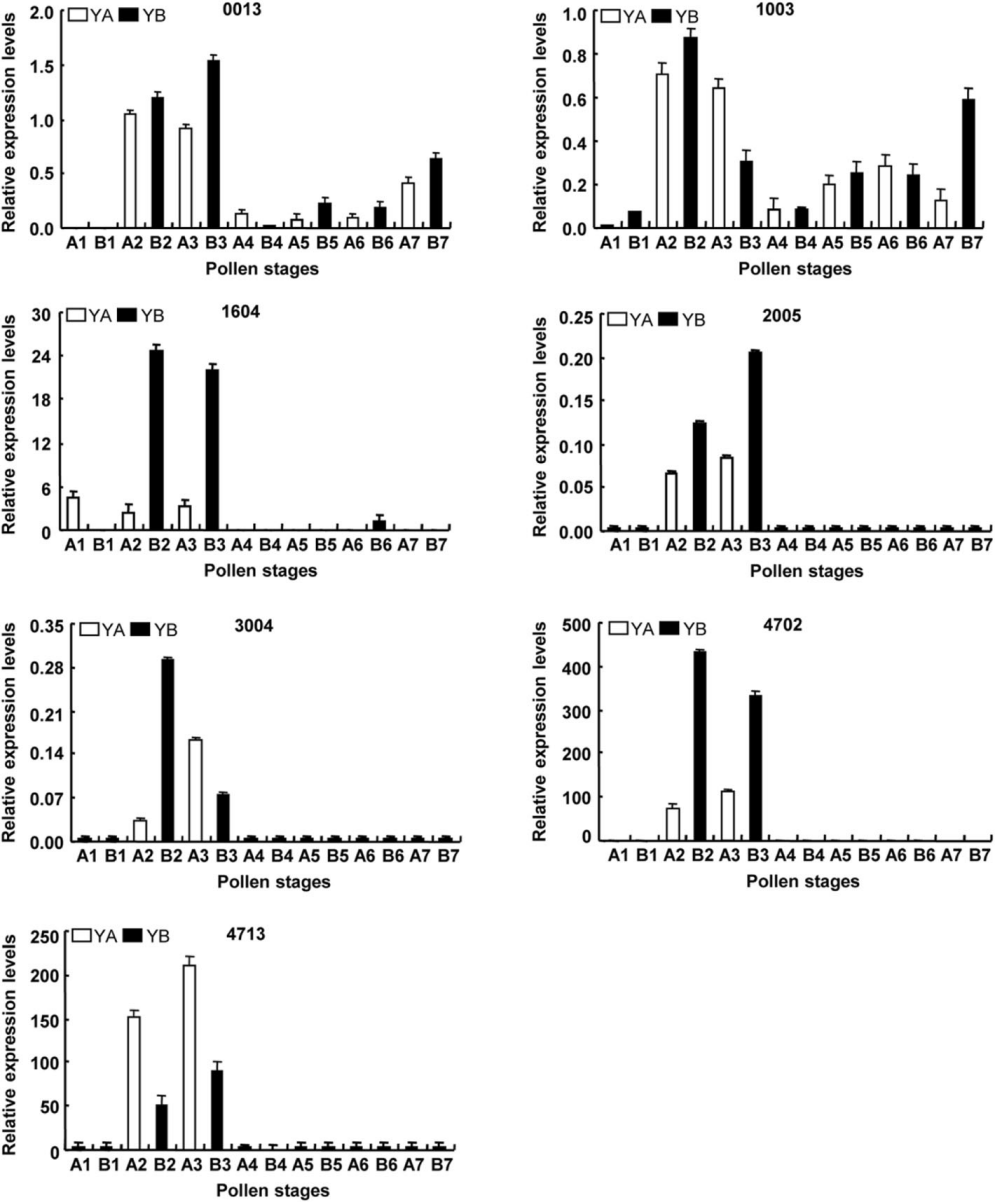
Real-PCR validation of different abundances of proteins at the mRNA level. A1-A7 and B1-B7 represent seven different development periods of the pollen on Yamian A and Yamian B, respectively

Seven coding genes that corresponded to differentially expressed proteins were selected to analyze in the mRNA expression levels by qRT-PCR to examine our 2-DE results and verify the differences in protein abundance at the transcriptional level (Fig. 6). The expression level of 1003 at the 2nd, and the 7th stages of the floral buds in Yamian A was lower compared to that at the same stages in Yamian B, but instead at the 3rd stage of the flower bud, and a notable difference in other periods between Yamian A and Yamian B was not observed. The expression level of 3004 at the 2nd stage of the flower bud in Yamian A was lower compared to that at the same stages in Yamian B, but instead at the 3rd stage of the flower bud. The expression levels of 0013, 1604, 4702 and 2005 at the 2nd and 3rd stages of the flower buds in were lower compared to those at the same stages in Yamian B. The expression level of 4713 at the 2nd and 3rd stages of the flower buds in Yamian A was higher compared to that at the same stages in Yamian B.

#### Comparative analysis between TDFs and DEPs

To study the abortive cause of YA-CMS, we performed a comparative analysis between TDFs and DEPs in terms of expression, functional annotation, GO and KEGG analyses. We found four interesting things. First, we found peroxidase-related annotations (T74 and 3003) between TDFs and DEPs; however, T74 was highly expressed only in the A2 period, while 3003 was highly expressed in the B3 period. Second, the expression of different TDFS or DEPs with the same annotation, such as the NAC domain, the NAC domain superfamily (T51 and T55), and the ribulose bisphosphate carboxylase, large subunit (4702 and 5720), was not consistent between Yamian A and Yamian B. Third, there was 1 identical cell component, integral component of membrane (GO:0016021), and 2 molecular functions, protein binding (GO:0005515) and ATP binding (GO:0005524), according to the GO analysis of TDFs and DEPs. Fourth, there were no identical KEGG pathways between TDFs and DEPs. These results indicated that the two study perspectives, transcriptomics and proteomics, were consistent and complementary and demonstrated that there might be a complex regulatory network between the genes and proteins derived from three different genetic backgrounds of *Gossypium bickii*, *Gossypium arboreum* and *Gossypium hirsutum* Linn in YA-CMS.

## Discussion

### Relationship between pollen abortion and the differences in ATP synthase in the YA-CMS and YB plants

ATP synthase is the key enzyme in the process of mitochondrial oxidative phosphorylation. Mitochondria ATP synthase belongs to the energy storage “F” type, which consists of two parts, the Fo and F_1_ regions. The Fo region is located within the inner membrane of plant mitochondria and functions as a proton channel. F_1_ is the active enzyme center and is composed of alpha, beta, and other subunits. The binding sites of beta subunits have the activity of catalytic ATP synthesis or hydrolysis [21].

Pollen development is a process of high energy consumption, and some or some gene products interfere with the function of mitochondrial FoF_1_-ATP synthase, which may lead to the abortion of pollen [22]. Many studies have shown that ATP synthase has a close relation with cytoplasmic male sterility. For example, Li et al.’s study of protein interactions in chili pepper indicated that the decreased activity and amount of ATP synthase affected the development of pollen and thus caused cytoplasmic male sterility [23]. The use of the SNP marker of the ATP synthase gene could simply, rapidly and easily identify the cytoplasmic male sterile line CMS-D8 [24]. The study results of *atp1* [25], *atp4* [26], *atp6* [27], *atp8* [28] and *atp9* [29] by researchers showed that these genes may be related to cytoplasmic male sterility in plants. Studies have also found that 29 mtDNA regions associated with CMS have been identified, and these recombinant chimeric genes are involved in the promoter region and part of the coding region of the ATP synthase subunit gene. Kong et al.’s RNA editing analysis of ATP synthase genes on the cotton CMS line H276A, maintainer line and restorer line showed forty-one RNA editing sites, and two new stop codons were detected and suggested the ATP synthase genes might be an indirect cause of cotton CMS [30]. Protein analysis in CMS of wheat showed ATP synthases could be associated with abnormal pollen grain formation and male sterility [31].

In this study, we found 3 ATP synthase-related proteins from the proteome, 2 ATPase, F1/V1/A1 complex, alpha/beta subunit, and nucleotide-binding domain (1604, 4713), and 1 ATPase, F0 complex, subunit D, mitochondrial (0013).

Among these proteins, 1604 was significantly upregulated in the sporogenous cell and microsporocyte stage of the YB plants but not in YA-CMS plants at the same stages. The expression of 0013 was significantly reduced in the microsporocyte stage of the YA-CMS plants compared with the YB plants in the same period. Spot 4713 was more significantly upregulated in the microsporocyte stage of the YA-CMS plants than in YB plants. Zheng et al. ‘s study found that ATP synthase beta subunit and ATP synthase D chain were downregulated in Male Sterile Mutant YX-1 anthers of Wolfberry [32]. Li et al.’s study found that ATP synthase beta subunit was not expressed in the wheat BNS male sterile line but was expressed in its transformation line [33]. These results were consistent with ours. Differential proteomics was studied with the upland cotton cytoplasmic sterile line 104-7A, maintainer line, and restorer line by Xu Qi, and the results found that ATP synthase beta subunit was expressed only in the restorer line, while there was no expression in the sterile and maintainer lines [34]. Wu et al.’s study found that ATP synthase D chain was downregulated in *Capsicum annuum* L. CMS anthers, but ATP synthase beta subunit was upregulated in the same material [35]. These findings were not consistent with ours results. According to previous ultrastructure observations, the sporogenous cell and microsporocyte stages of the YA-CMS plants both contained numerous abnormal mitochondria. The above results showed that the downregulation of ATPase, F1/V1/A1 complex, alpha/beta subunit, nucleotide-binding domain (1604) and 1 ATPase, F0 complex, subunit D, mitochondrial (0013) led to internal energy metabolism disorder, caused large mitochondrial abnormal disintegration, and then affected the development of anther, ultimately causing male sterility in the YA-CMS plant. Additionally, the disagreements in the up- and down-regulation of ATP synthase and its subunit from different male sterile lines in different plants, and even the same kind of plant but different genotypes, may be caused by their self-different abortion mechanisms; these different sterility mechanisms are still not well understood and warrant further research.

### Relationship between pollen abortion and differences in UGT in YA-CMS and YB plants

UGT are the major glycosyl transferase in plants. These proteins can transfer the glycosyl groups of the activated donor molecule (mainly uridine diphosphate glucose) to the receptor molecule (including secondary metabolites, such as flavonoids, phytohormones, such as cytokinins, and herbicides and insecticides), thereby regulating the location of the receptor molecule in the cell and its biological activities such as solubility and transport in organisms [36, 37]. UGT plays an important role in regulating glycosylation and energy storage of secondary metabolites in organisms, endogenous hormone activity and toxicity relief of exogenous toxins [38, 39]. In this study, three different primers (E2M7, E9M16 and E15M3) were used to amplify the four differences in the highest consistency with the *UGT* gene (T1, T12, T26, and T27) in the buds of the YA-CMS plant during the peak period of abortion (sporogenous cell proliferation stage, microspore mother cell stage and meiosis stage) and after the abortion stage (tetrad stage, first nuclear stage and pollen maturation stage) of microspores. The allogenic fragments were not amplified in the maintainers of the same period. This suggests that the *UGT* gene may play a role in the peak period of microspore abortion of the cotton male sterile line Yamian A and may be related to the microspore abortion of Yamian A, but this hypothesis still needs further experiments to verify.

### Relationship between pollen abortion and the differences in ribosomal proteins in YA-CMS and YB plants

The ribosome is a protein-nucleic acid complex enzyme system [40]. As the main site of protein synthesis in cells, the integrity of the ribosome structure and the coordination of the quantity of each component are necessary conditions to ensure the effective and correct synthesis of protein [41]. Although it is generally believed that these ribosomal proteins play an important role in protein synthesis, more ribosomal proteins have been reported to have many other functions. For example, they can play a role in the regulation of cell apoptosis, proliferation, development, and malignant transformation by participating in transcription, RNA processing, DNA repair and replication [39]. Zhou et al. found that ribosome proteins were essential for anther development and male sterility in sterile buds when they studying the genetic male sterile line ‘AB01’ in Chinese cabbage [5]. This study indicates that there is a certain relationship between ribosomal protein and plant male sterility.

The 60S ribosomal protein L13a-4-like (T2) of *G. austral* was isolated, and expressed only at the peak of microspore abortion of cotton cytoplasmic male sterile line Yamian A, but no expression was observed during microspore developmen and other periods in the male sterile line Yamian A and the whole anther formation period of the maintainer line. This result suggests that the 60S ribosomal protein L13a-4-like may be involved in the development of microspores in the male sterile line, which is related to the abortion of microspores in male sterile lines, but this hypothesis still needs further experiments to verify.

### Relationship between pollen abortion and the differences in NAC transcription factors in the YA-CMS and YB plants

NAC transcription factors are one of the largest families of transcription factors peculiar to plants. These factors have many functions, and they are widely involved in the formation of lateral roots, secondary walls, shoot apical meristem, senescence and flowering of plants, as well as the response to abiotic and biological stresses [42]. Chen et al. found that 9 NAC transcription factor genes were downregulated and 6 NAC transcription factor genes were upregulated in sterile buds when they studied CMS in Wucai [43].

Our study results showed that three TDFs, T51, T52, and T55, had the same annotation NAC domain; NAC domain superfamily. T55 was amplified from the mixed buds of the peak period of abortion (sporogenous cell proliferation stage, microspore mother cell stage and meiosis stage) of YA-CMS by using selection primers E7M3 but was not amplified in the buds of the cotton maintainer line in corresponding period. T51 and T52 were amplified from the mixed buds of microspore development tetrads, monocyte and binucleate pollen grains and mature pollen grains of the cotton maintainer line YB by using the selection primer E7M2 but were not amplified in the buds of the sterile line in the corresponding period. In terms of cell morphology, at this stage, the pollen sac of the male sterile line anthers contracted and decreased after microspore mother cells disintegrated completely. Then, the tapetum cells elongated radially and filled the pollen sac during the tetrad formation of fertile anthers and finally formed pollen sacs without pollen grains. This result suggests that pollen abortion of CMS lines may be caused by mutation or silencing of the NAC transcription factor gene, but this hypothesis still needs further experimental verification.

### Relationship between pollen abortion and the differences in ribulose bisphosphate carboxylase in YA-CMS and YB plants

Ribulose bisphosphate carboxylase is widely distributed in the organelles of photosynthesis. It is a key enzyme for fixing CO_2_ in plant photosynthesis and participates in the photorespiration pathway of plants. Ribulose bisphosphate carboxylase is composed of 8 small subunits (12–18 kD) encoded by nuclear genes and 8 large subunits (50–60 kD) encoded by chloroplasts. The small subunits have regulatory functions, and the enzyme activity locates on the large subunits. Kurepa J and Smalle J A found that the oxidative stress caused by promoting the generation of superoxide anion induced the formation of covalently linked ribulose-1,5-bisphosphate carboxylase/oxygenase large subunit dimer, and its formation coincided with the loss of chloroplast function when they studied the effects of oxidative stress on tobacco [44].

Current studies on cytoplasmic male sterility in many plants have shown that ribulose bisphosphate carboxylase is related to fertility. Chen et al. showed that the expression of the ribulose bisphosphate carboxylase subunit in two stages of the wheat cytoplasmic-nuclear interaction male sterile line was significantly downregulated, and this result suggested that energy metabolism might be closely related to anther development [45]. Liu et al. found that the activity of ribulose bisphosphate carboxylase in cytoplasmic male sterile lines of maize, sorghum, rice, wheat, and tobacco was higher than that in their corresponding maintainer lines, indicating that there was a certain relationship between ribulose bisphosphate carboxylases or their cytoplasmic male sterility in plants [46]. Ren Yan also identified five ribulose bisphosphate carboxylase or its large subunits in the differential proteome analysis of anthers of double recessive genic male sterile lines and fertile lines of *Gossypium hirsutum* Linn [47].

Two ribulose bisphosphate carboxylase, large subunit spots (4702, 5720) were found in our study. The spots (4702, 5720) on the 2-DE diagram show that the molecular weight is the same, but the isoelectric point is not the same: one is acidic, and the other is alkaline. The acidic large subunit was expressed only in the critical period of abortion of the maintainer line, while the alkaline large subunit was only expressed in the sterile line at the critical period of abortion. The difference between the 2 ribulose bisphosphate carboxylase, large subunit spots between the sterile and maintainer lines may be caused by the differing degrees of reactive oxygen species, and this may be related to anther fertility of cytoplasmic male sterility, though the specific mechanism needs further study.

### Relationship between pollen abortion and the differences in heat shock protein in the YA-CMS and YB plants

Heat shock protein is a kind of stress protein induced and synthesized by organisms under the influence of adverse environmental factors such as high temperature, hypoxia, starvation, and heavy metal ions. It can improve the heat resistance of cells and has the functions of molecular chaperone and regulation. At present, heat shock protein has become the focus of molecular biology research, and there are some reports on male sterility. Heat shock protein *HSP70* gene transcription was blocked in the sterile line, which caused abnormal cell meiosis, resulting in the number of anther mitochondria in the sterile line, and then pollen development could not obtain sufficient energy, resulting in pollen abortion [48, 49]. Zeng et al. also found heat shock protein 22 kDa in anther differential proteomics of the soybean cytoplasmic male sterile line NJCM2A and speculated that it might lead to abnormal mitochondrial development, thus resulting in inadequate energy supply for pollen development and eventually abortion [50]. Su et al. found that six BoHSP70 genes were highly expressed in the binuclear-pollen-stage buds of a male fertile line compared with its near-isogenic sterile line when they studied the HSP70 family genes in cabbage [51].

In this study, heat shock protein Hsp20 (2005) was found in the buds of YA-CMS and YB during the critical period of abortion, and its expression in YB was higher than that in YA-CMS. Our results were similar to the results of Su et al. The difference in expression of heat shock protein Hsp20 (2005) between sterile lines and maintainer lines indicates that 2005 may be related to anther fertility of cytoplasmic male sterility.

## Conclusions

**Fig. 7:**
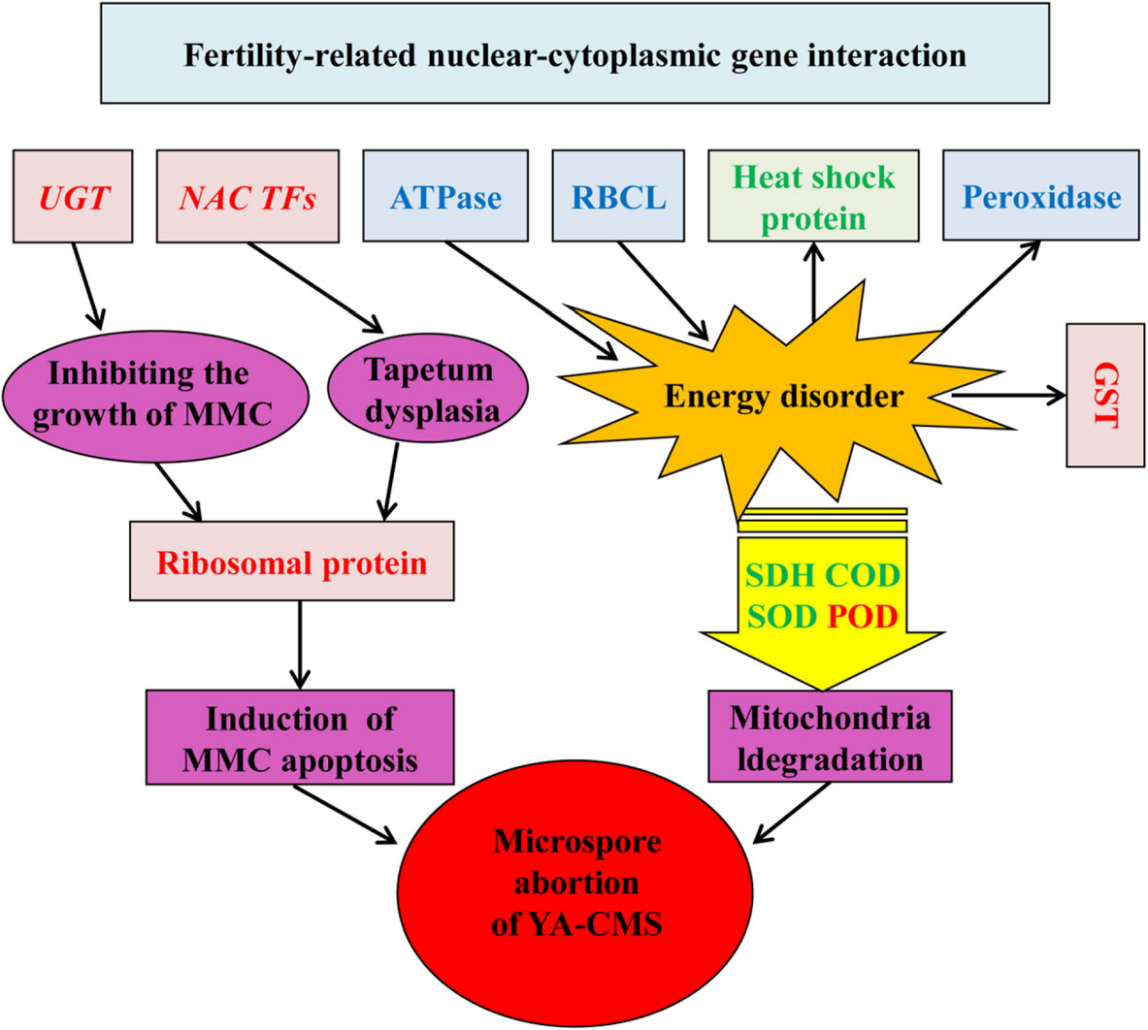
Hypothetical interaction network of microspore abortion in the cytoplasmic male sterile line Yamian A. Red font: upregulated expression; green font: downregulated expression; blue font: upregulated and downregulated expression; MMC: microspore mother cells; POD: peroxidase; SOD: superoxide dismutase, COD: cytochrome oxidase; SDH: succinic dehydrogenase

Combining all results of the transcriptome, proteome and early cytological, physiological and biochemical studies of the cytoplasmic male sterile line Yamian A and its maintainer line Yamian B in cotton, we speculated that there might be connections among UGT, NAC transcription factors (NAC TFs), ATPase, ribulose bisphosphate carboxylase, large subunit (RBCL), glutathione S-transferase (GST), heat shock protein, peroxidase, and ribosomal protein regarding the cytoplasmic male sterility of Yamian A (Fig. 7). However, the occurrence of cytoplasmic male sterility has certain temporal and spatial specificity. Further studies are still needed to determine the exact nature of the full mechanism underlying cytoplasmic male sterility in Yamian A.

## Methods

### Plant materials

Both the cytoplasmic male sterile (CMS) line Yamian A and its maintainer Yamian B are from the cotton breeding group of Shanxi Agricultural University.

The breeding process of the CMS Yamian A and its maintainer Yamian B was as follows: the diploid *Gossypium arboreum* (A_2_A_2_) was used as the female parent, the wild *Gossypium bickii* (G_1_G_1_) was used as the male parent to hybridize into the allodiploid (A_2_G_1_), the hybrid chromosome was doubled, the new heterotetraploid (A_2_A_2_G_1_G_1_) was synthesized, and the tri-specific hybrid (AAGD) was synthesized by hybridizing the heterotetraploid (A_2_A_2_G_1_G_1_) with the cultivated tetraploid *Gossypium hirsutum* Linn (AADD)_1_ [52–54]. The natural mutant cytoplasmic male sterile material found in the progeny of tri-specific hybrids was used as the source of sterility, and the BC_6_ cotton cytoplasmic male sterile Yamian A was obtained through interspecific hybridization and continuous backcrossing with upland cotton Yamian B as the recurrent parent. Yamian A has the cytoplasm of *Gossypium arboreum* and the genetic background of wild *Gossypium bickii* in Australia. Yamian B is the homotype maintainer of Yamian A.

We analyzed the flower organ morphology, fertility performance, restoration and conservation relationship, cytology, physiological and biochemical, and Random Amplification Polymorphic DNA (RAPD) of Yamian A. The main study results were as follows:

The anther of Yamian A was thin, dark brown, shriveled and not dehiscent, and no pollen dispersed, but its pistil development was normal. The anther in its maintainer Yamian B was plump, and milky yellow, and the pollen is scattered and full of the whole anther after cracking (Fig. S4) [16]. The fertility of Yamian A was not affected by the environment, and the sterility was stable; Yamian A has a 100% rate for both sterile plants and degree of sterility, and the outcrossing rate was high, which was distinctly different from that of the *Gossypium arboreum* cytoplasmic male sterile line, reported to be susceptible to the environment [55, 56]. Both upland cotton and island cotton could be used as maintainers of Yamian A. The recovery materials 10N93R and 10N91R (introduced from the cotton Institute of Shanxi Academy of Agricultural Sciences) transferred from 0 to 613-2R had good recovery ability for Yamian A. The abortion of microspores of Yamian A was caused by delayed development of tapetum cells (Fig. S5) [16], which was different from the abortion methods summarized by previous studies that were caused by excessive hypertrophy or premature disintegration of tapetum cells; the activities of peroxidase and so on were related to the male sterility of Yamian A [57]. The source of the material and mitochondrial RAPD analysis indicated that the sterile cytoplasm of Yamian A was different from that of the existing *Gossypium harknessii* cytoplasmic male sterile line (Ha A) [58] and jin A [59] (Fig. S6).

These results indicate that Yamian A is a new sterile material with the cytoplasm of *Gossypium arboreum* and the genetic background of Australian wild *Gossypium bickii*, and Yamian A is novel for study.

The cotton CMS line Yamian A (YA-CMS) and its maintainer Yamian B (YB) were planted in the experimental field of Shanxi Agricultural University, Taigu, Shanxi, China, during the natural growing season. Referring to Hou’s method [60], based on the observation and analysis of a large number of cotton anther morphology and cytology, we determined the stable correlation between bud transverse diameter (BTD) and pollen development stages on YA-CMS and YB, and the flower buds were divided into seven consecutive grades (Table S3). Stage 1 (Sporogonium stage, BTD ≤ 1.50 mm): the buds were the normal development and before microspore abortion stage; Stage 2 (Sporogenous cells stage, 1.50 < BTD ≤ 2.16 mm), 3 (Microsporocyte stage, 2.16 < BTD ≤ 2.60 mm) and 4 (Meiosis stage, 2.60 < BTD ≤ 4.60 mm): the buds were the fertility transformation and middle microspore abortion stage, stages 2 and 3were the key stage of pollen abortion; Stage 5 (Tetrad stage, 4.60 < BTD ≤ 5.90 mm), 6 (First nuclear stage, 5.90 < BTD ≤ 9.93 mm) and 7 (Pollen maturation stage, BTD > 9.93 mm): the buds were entirely abortive and after the microspore abortion stage [16].

At the anthesis, the buds of 7 different stages were harvested separately from more than 55 plants of YA-CMS and YB, and the mixed buds of every stages from each line were weighed in 2 g packages, then rapidly frozen as packed materials with liquid nitrogen and preserved at − 80 °C for later experiments.

The buds of the before, middle and after microspore abortion stages of Yamian A and Yamian B were collected for transcriptome research; the buds of the key stage of pollen abortion (Stage 2, 3) were collected for proteomics research; the buds of the seven different development periods were used to perform analysis by qRT-PCR. An individual hybrid dynamic sampling method was used in the sampling process to ensure that each sample has the same genetic background and growth period.

### Transcriptome analysis

The total RNA of the buds collected for the transcriptome research was extracted by the EASYspin Plus Plant RNA Kit RN37 (Aidlab Biotechnology) and cDNA synthesis by the M-MLV RTase cDNA Synthesis Kit (TaKaRa Company). cDNA-AFLP analysis was performed and slightly changed as described previously [61]. Each sample was used with three technical replicates. The differentially expressed band’s sequences were analyzed with DNASTAR software and the BLAST instrument in the latest *G. austral*, *G. arboreum* and *G. hirsutum* genomic databases of CottonGen (https://www.cottongen.org/).

### Proteomics analysis

Protein isolation, 2-DE, image analysis, tryptic digestion and identification of differentially expressed proteins were performed as described previously with some modifications [62]. Each sample was used with three technical replicates. The mass spectrometry data of differentially expressed proteins were identified by MASCOT and PEAKS 6.0 software, and their sequences were analyzed in the *G. raimondii, G. austral*, *G. arboreum* and *G. hirsutum* genomic databases of CottonGen (https://www.cottongen.org/) [63]. STRING 11.0 (http://string-db.org/cgi/input.pl) was used to construct a protein-protein interaction network of differential proteins with *G. raimondii* as the reference species.

### Quantitative real-time PCR verification

Total RNAs extraction, reverse transcription and qRT-PCR from the buds of seven different development periods of both the fertile and sterile plants were performed using EASYspin Plus Plant RNA Kit RN09 (Aidlab Biotechnology), PrimeScript® RT Master Mix Perfect Real-Time and DRR820ASYBR® Premix Ex Taq™ II (Tli RNaseH Plus) (TaKaRa), respectively, according to the manufacturer’s instructions. The relative expression of the target genes was calculated with the 2^-△△Ct^ method [64]. Primers for qRT-PCR analysis are shown in Supplementary Table S4. There were three biological replicates with three technical replicates per sample.

## Supplementary Information

**Additional file 1: Figure S1.** Selective amplified products of several primer pairs. 1, 3, 5 and 2, 4, 6 represent anther of before, middle and after microspore abortion stages of Yamian A and Yamian B. A, B, C, D, E represents different kinds of expressed bands between Yamian A and Yamian B.

**Additional file 2: Figure S2.** 2-DE images of flower bud proteins in the sporogenous cell and microsporocyte stages from YA-CMS and YB. A2: sporogenous cell stage of YA-CMS; A3: microsporocyte stage of YA-CMS; B2: sporogenous cell stage of YB; B3: microsporocyte stage of YB.

**Additional file 3: Figure S3.** Parts of TDFs on cDNA-AFLP. 1, 3, 5, and 2, 4, 6 represent anther of before, middle and after microspore abortion stages of Yamian A and Yamian B.

**Additional file 4: Figure S4.** Anther morphology in CMS line Yamian A and its maintainer Yamian B [16].

**Additional file 5: Figure S5.** Microstructure of the stamen in CMS line Yamian A and its maintainer Yamian B [16].

**Additional file 6: Figure S6.** Polymorphism of the E89397 primer amplified on Yamian A, Jin A, and Ha A.

**Additional file 7: Table S1.** Important types of gene differential expression.

**Additional file 8: Table S2.** TDF sequences on cDNA - AFLP.

**Additional file 9: Table S3.** Bud developmental stages in cotton [16].

**Additional file 10: Table S4.** Primers used in qRT-PCR.

## Data Availability

The genome databases of *G. raimondii, G. austral*, *G. arboreum* and *G. hirsutum* were downloaded from the CottonGen (https://www.cottongen.org/). All the data supporting the results in this article are included in the present and the additional files.

## Abbreviations

CMS: Cytoplasmic male sterility
DEPs: Differentially expressed proteins
UGT: UDP-glucuronosyl/UDP-glucosyltransferase
RBCL: Ribulose bisphosphate carboxylase, large subunit
GST: Glutathione S-transferase
YA-CMS: Yamian A CMS
YB: Yamian B
qRT-PCR: Quantitative real-time PCR
cDNA-AFLP: cDNA amplified fragment length polymorphism
GO: Gene ontology
KEGG: Kyoto encyclopedia of genes and genomes
TDFs: Transcript-derived fragments
