# Supplementary figures and images for: Transcriptomic and proteomic analyses of a new cytoplasmic male sterile line with a wild Gossypium bickii genetic background
Source: BMC Genomics. 2020 Dec 2;21:859. doi: 10.1186/s12864-020-07261-y (PMC7709281; doi:10.1186/s12864-020-07261-y)

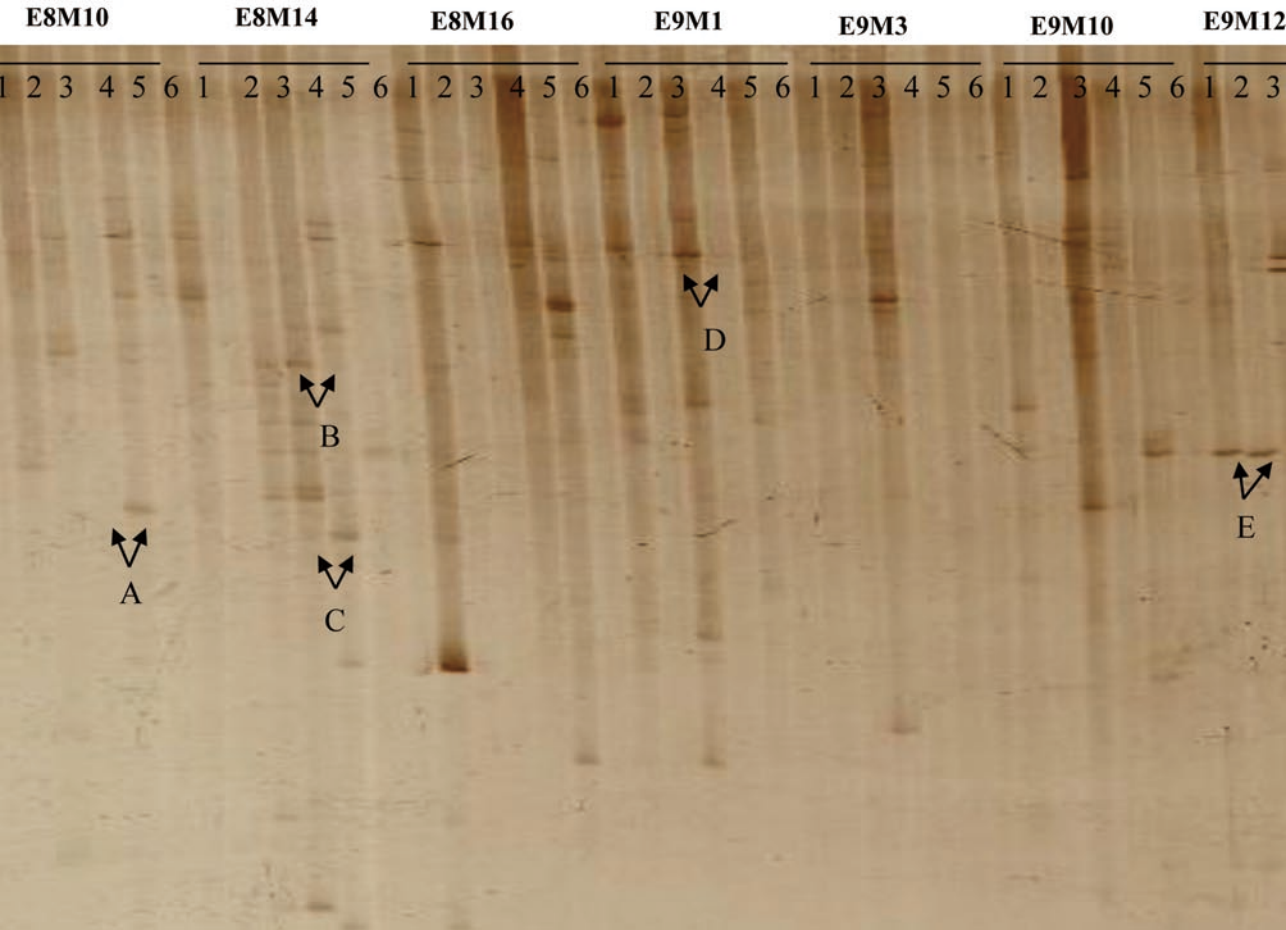

Supplement: Supplementary file 1 — Additional file 1: Figure S1. Selective amplified products of several primer pairs. 1, 3, 5 and 2, 4, 6 represent anther of before, middle and after microspore abortion stages of Yamian A and Yamian B. A, B, C, D, E represents different kinds of expressed bands between Yamian A and Yamian B. [file 12864_2020_7261_MOESM1_ESM.pdf]

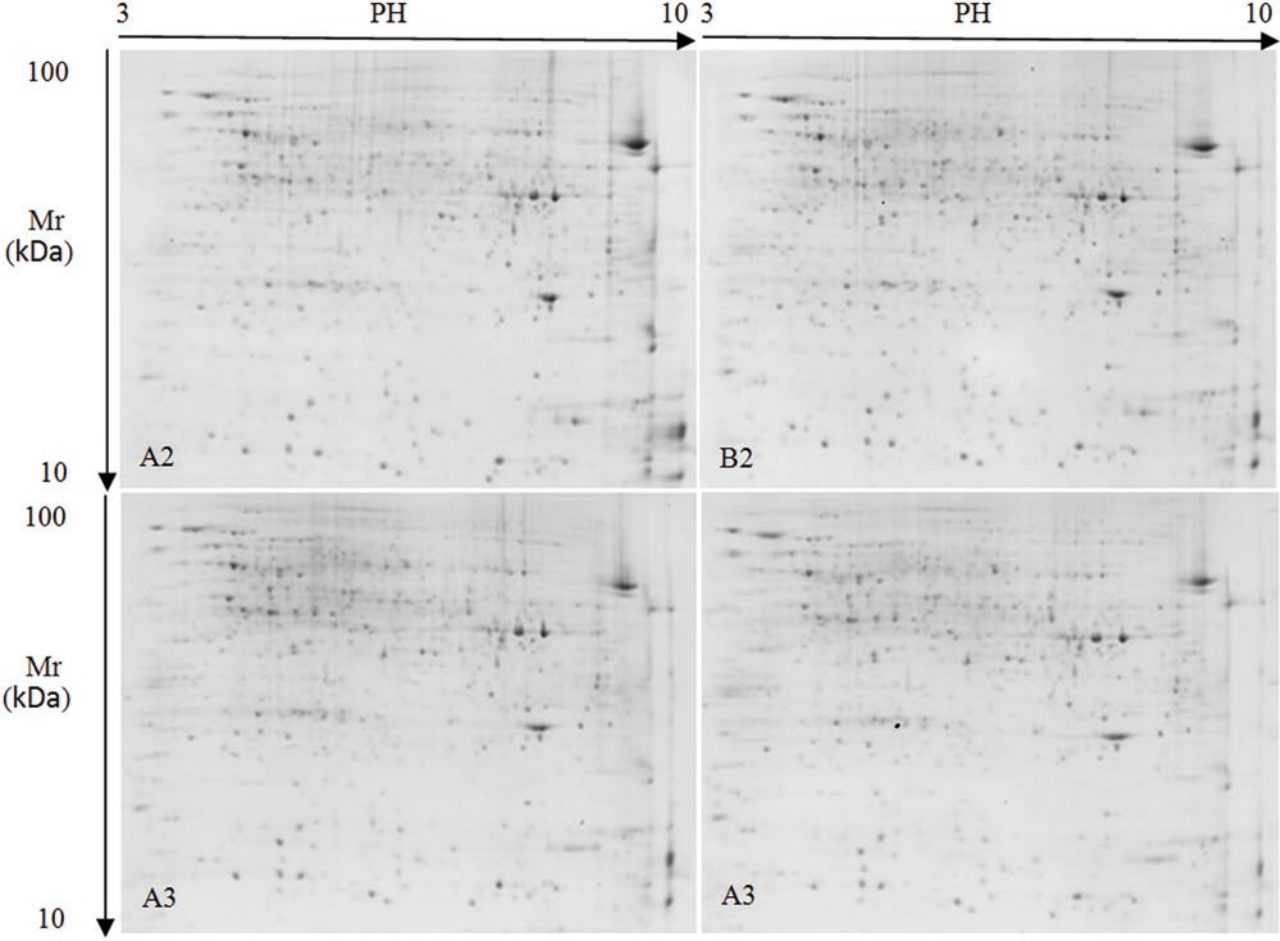

Supplement: Supplementary file 2 — Additional file 2: Figure S2. 2-DE images of flower bud proteins in the sporogenous cell and microsporocyte stages from YA-CMS and YB. A2: sporogenous cell stage of YA-CMS; A3: microsporocyte stage of YA-CMS; B2: sporogenous cell stage of YB; B3: microsporocyte stage of YB. [file 12864_2020_7261_MOESM2_ESM.pdf]

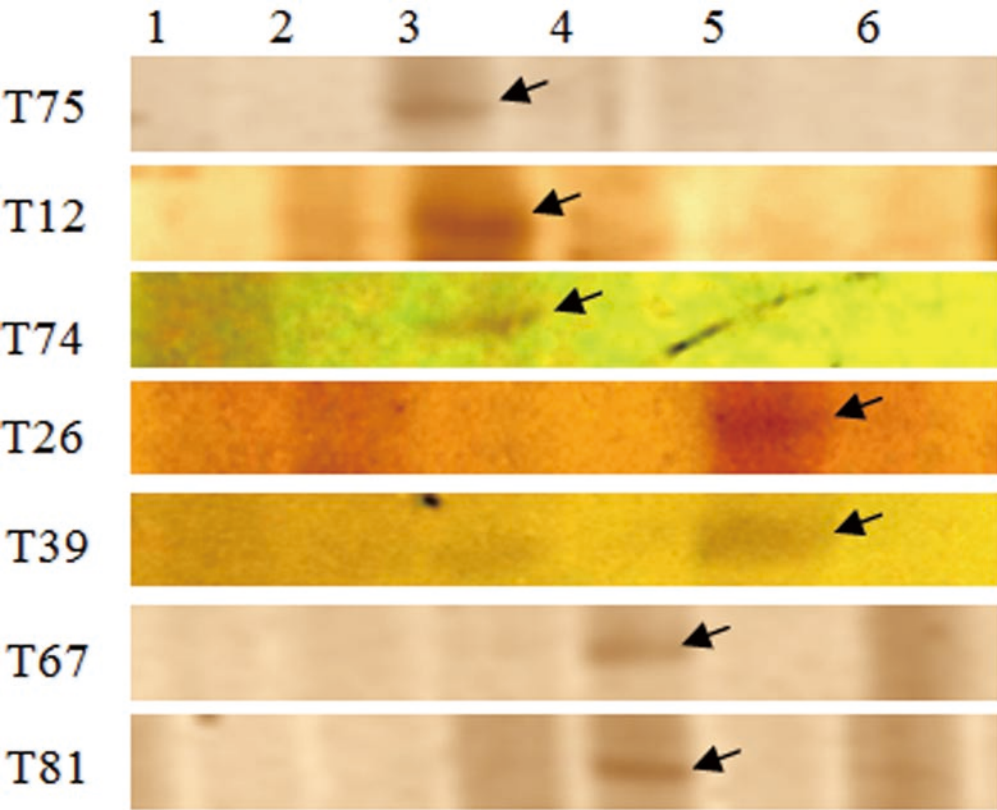

Supplement: Supplementary file 3 — Additional file 3: Figure S3. Parts of TDFs on cDNA-AFLP. 1, 3, 5, and 2, 4, 6 represent anther of before, middle and after microspore abortion stages of Yamian A and Yamian B. [file 12864_2020_7261_MOESM3_ESM.pdf]

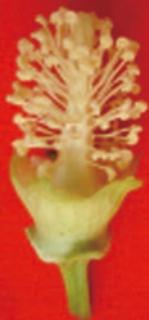

Yamian B

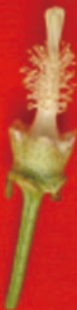

Yamian A

Supplement: Supplementary file 4 — Additional file 4: Figure S4. Anther morphology in CMS line Yamian A and its maintainer Yamian B [16]. [file 12864_2020_7261_MOESM4_ESM.pdf]

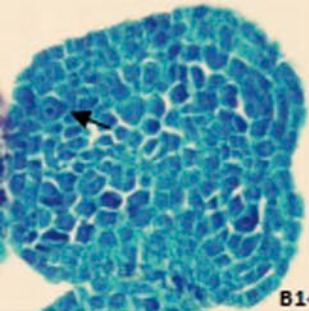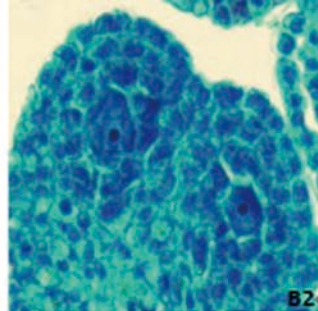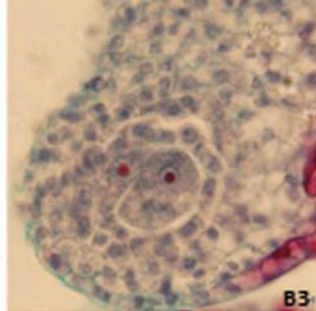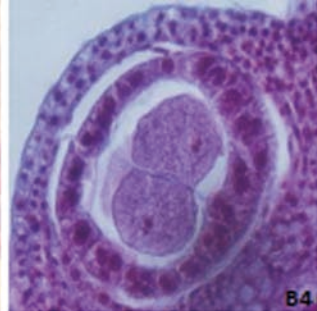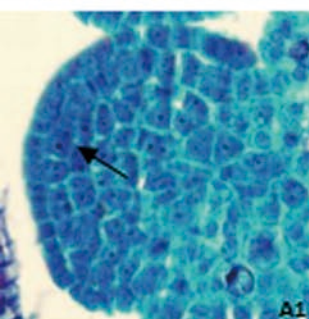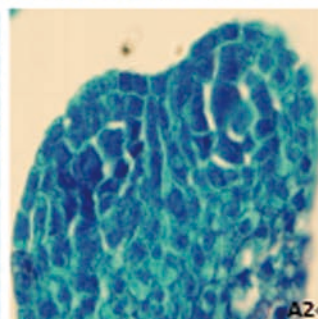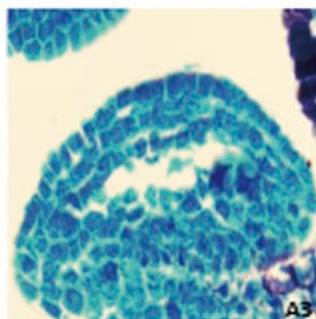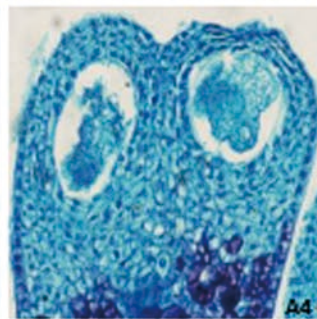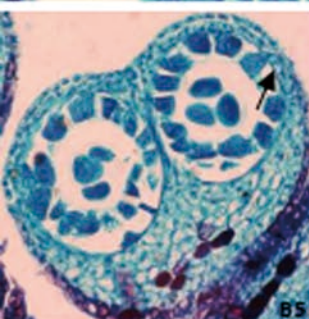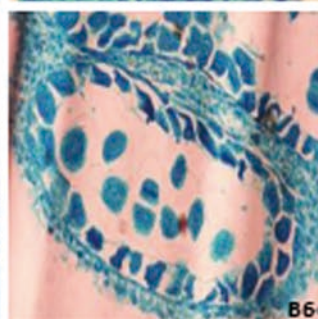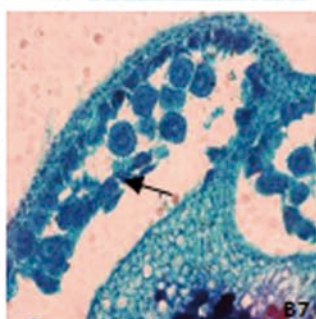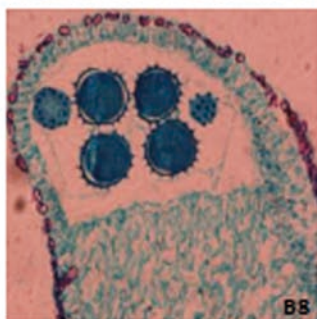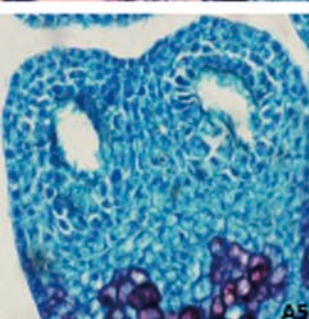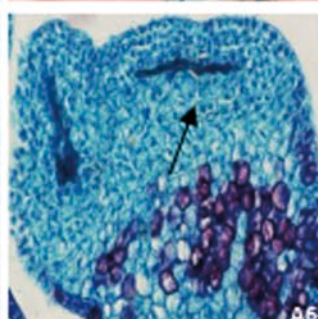

Supplement: Supplementary file 5 — Additional file 5: Figure S5. Microstructure of the stamen in CMS line Yamian A and its maintainer Yamian B [16]. [file 12864_2020_7261_MOESM5_ESM.pdf]

Yamian A

Jin A

Ha A

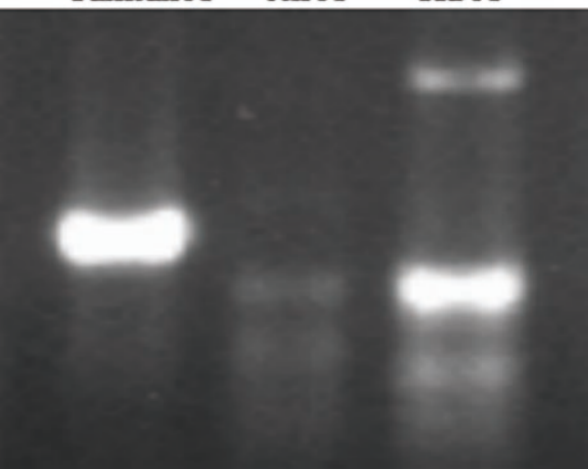

Supplement: Supplementary file 6 — Additional file 6: Figure S6. Polymorphism of the E89397 primer amplified on Yamian A, Jin A, and Ha A. [file 12864_2020_7261_MOESM6_ESM.pdf]
