# Supplementary material for: Transcriptomic and proteomic analyses of a new cytoplasmic male sterile line with a wild Gossypium bickii genetic background
Source: BMC Genomics. 2020 Dec 2;21:859. doi: 10.1186/s12864-020-07261-y (PMC7709281; doi:10.1186/s12864-020-07261-y)
Supplement: Supplementary file 7 — Additional file 7: Table S1. Important types of gene differential expression. [file 12864_2020_7261_MOESM7_ESM.pdf]

Table S1. Important type of gene differential expression.

Type	S1	F1	S2	F2	S3	F3	Total bands	primer number
1	+	-	-	-	-	-	12	8
2	-	-	+	-	-	-	100	36
3	-	-	-	-	+	-	42	17
4	-	+	-	-	-	-	17	8
5	-	-	-	+	-	-	20	5
6	-	-	-	-	-	+	5	4
7	+	-	+	-	-	-	12	4
8	-	-	+	-	+	-	63	11
9	+	-	-	-	+	-	20	6
10	-	+	-	-	-	+	7	1
11	-	+	-	+	-	-	14	3
12	-	-	+	+	-	-	20	6
13	-	-	+	+	+	-	17	3
14	+	+	+	-	-	+	5	1
15	+	+	+	-	-	+	15	1

S1, S2, S3 and F1, F2, F3 represent anther of before, middle and after microspore abortion stage of Yamian A and Yamian B respectively; +: with a band, -: no band.
