# Supplementary material for: Transcriptomic and proteomic analyses of a new cytoplasmic male sterile line with a wild Gossypium bickii genetic background
Source: BMC Genomics. 2020 Dec 2;21:859. doi: 10.1186/s12864-020-07261-y (PMC7709281; doi:10.1186/s12864-020-07261-y)
Supplement: Supplementary file 8 — Additional file 8: Table S2. TDF sequences on cDNA - AFLP. [file 12864_2020_7261_MOESM8_ESM.pdf]

Table S2. TDF sequences on cDNA - AFLP.

Name	Sequences
T1	TTAACCCATTGAAAGAACTTGTAGAACCGTCTTCCATAAGACAAAGAGCAGCTTTCTTCTTCAAATCCATAGCTCTCTTTCTGATATCCTTTCCTTCCATATCCACCATTAGTCTTTTTATTGTTCTTTCTATATTCCCTCTTTCTA<br>GATTCTGCAATTCCAAGCCTATTTTCCAAACATTACATATGTAACTTGTGTTCAAGAGTTGGTCGCCAAAGAAAGGTCTGCATAGCATTGGTACCCCTTCACAAATGCTCTCAATGGTTGAATTC
T2	GAATTCAACAACACAAAACTTCAACAATAACCTCGAGTAGTCACTTTGAAACTAACATAAAGGTATCCATCCTAAGTCAAATTTCAAGCTTATACCTTACTCAGGACTCATCGTCAGAGGTCGGATGGAACCACTATGACA<br>CTATCAAGGAGCTTGAAAGGAAGAGGAAGGAAAGAGCTCAAGTGGCATACGAGAGAAGGAAGCAACTCACTAAGCTCAGGGTTAA
T3	TTAACCCCCAAAATTTTTATTTCCCTCATCTCAATTTTCCATCCTTTGTCCACACATCTGTTGACTGTGTTGGACCATGTGCACTTCAATTCTTTTCAATGTGTTGAATTC
T4	TTAACCCATTGAAAGAACTTGTAGAACCGTCTTCCATAAGACAAAGAGCAGCTTTCTTCTTCAAATCCATAGCTCTCTTTCTGATATCCTTTCCTTCCATATCCACCATTAGTCTTTTTATTGTTCTTTCTATATTCCCTCTTTCTA<br>GATTCTGCAATTCCAAGCCTATTTTCCAAACATTACATATGTAACTTGTGTTCAAGAGTTGGTCGCCAAAGAAAGGTCTGCATAGCATTGGTACCCCTTCACAAATGCTCTCAATGGTTGAATTC
T5	GAATTCAACCAAGCGCGGGTAAACGGCGGGAGTAACTATGACTCTCGTAGACTGCGTACCAATTACCTCGTAGACTGCGTACCAATTACCTCGTAGACTGCGTACCAATTAATATTTGGGTTTTGTAATGGGTTTGGGTAGAT<br>GTAAATGGGTCATGGGTTAA
T6	TTAACTCTGAGAGCTTGTGTTTGTACATGTAAATTACTAAGCTTAGACATCGAGAACTACTATTATGAGCAGTGTAATATTTATGGAAGGAAAACCTGTTTGCTTGTTGGCAGTTGAATTC
T7	GAATTCAACAATGAAAATTTGAAGAAAAAAAATGTAAAAAGAAAAGGGGTTTGAGGGGTAATATCTGAGTTAA
T8	GAATTCAACTAATACAATATATAACGGACAAAAAAATTGGACAAAACTCACACAAAATATATATGTACATTGTGTGTTGAGAAGAGAGTCGGGAGTTAA
T9	GAATTCAACTGCCAACAAGCAAACAGGTTTTCCTTCCATAAATATTACACTGCTCATAATAGTAGTTCTCGATGTCTAAGCTTAGTAATTTACATGTACAAACACAAGCTCTCAGAGTTAA
T10	TTAACTCTATGGGCTTTGGGTTATGACAAATCGTTGATATTTTATGCATATCGGCTAATGTAAGCAACGTTGAATTC
T11	GAATTCAAGCCAAGTGACCACAAAGGGCCATTCTACTACGTTAA
T12	GAATTCATGCATGGAAAGCATCACCATGGGAGTACCAATAGGGGCTTGGCCTGCGCATTCAGATCAACCAAGAAACGCAGTACTAATCACAAAGTTGCTCAAACTCGGCATTACCATCAAGGACTGGGCACGTCGAGATG<br>AAGTAGTGACAGCAGCGGTAGTTGAAGATGCTGTGAAACGTTTGATGGCTTCAAAGGAAGGAGATGAGATAAGGAGGAGAGCGGCAGAAGTAAGTGGTGCGGTGCGCCAATCGGTGGCTGAAGGTGAGGTTTACCGTAAG<br>GAGTGGGACTCCTTCATTACTCATATCATTAGGTAGGTTGTATCATTTATTATTATCAAACTACTGCCACATGATTATTTTCCTTCAAGTATGTGTTAA
T13	TTAACACCCTGTACACTAAGCATCAACTTGAGAAGCAGGCAAGCAAGGAAAAGATATCATTTAGCCGCTTGCAAGTCCATGAGATTGCTGCATTTGTACTAAATTCATCTGGGCATTACGAGGCAATCACCCGAAACTGCTC<br>CAACTACTACTTGTCTGCTGAATCTGTGGCTTTGTTTACCGATCGTCTCCCAAGCCAACCAAGCTACATCGTCGGGCAGATTGTGCACATCGAGCGTCAAACTGTGAAGCCACTGCTGTCTTCATCAACTAGACCTGATCGTA<br>GCAGGGTGGATCCTGCAGAGCAACTAACATCTAACACCATGAATTC
T14	GAATTCATGAGCTATGGCATCGGTGTGATGTGATATGTGATTTCCGTGTAAGACCATATCTGGGATATGGCATCGGTGTGATATGTGATTTCCGTGTAAGATCATATCTGGGATATGACATCGGTGTGATATGTGTTTCTGTGTA<br>AGACCATAGTTGGGCTATGGCTTCGGTGTATGATATGTGACAATGTGCAAGACCATAGTTATACTATGGCAATGTGAAAATGAAGTACTCAATTTCGTAAAATGTTCTCTAATTTGACAAATTGTGGTAAGTGTTAA
T15	TTAACACGATCGCGAATCTCGACGCGTCTGGTCAAACTCTGGGTCGCCGCAATGGGACGATGACCTTTATGGAGGAGCTCGTCAAACTGCAAAAACCACCGAAATCTTCCTCCAAAGCTGACATTCAGAAATCTCGCGCTC<br>CGATCATCTGGATCGAGGGGACGGCCGGCGTCGGGAAATCTGCTCTGGCGAGGAATTTCTTTCGTCTTGCTAAGGTCAACGTGCCGACAATTTTCATCGCCGCTGAGGCCACGGACCGCAGTCGACCGTTTGGCATTTGGAC<br>GCACATTCTGGAGGAAATCTTGGGCCTCACGAAGGACATGAATTC
T16	TTAACACCGTGGGGGATGTCATGTCGCGGATCTGGCTGTGGCGGGCGTGTGTGGTGGGGGTGGCTTTGGCGCTGCCCGTGGCGGGGGCGATGGCGCAGGTGGATACAAGCGGGGCCACGACGGGCGCGCAGACCGTACCGG<br>CGGTCGCCACCATGGCAGGTGGCAGCAATATCTACGATGCCTCCGTGGCAGCCCTGACCAAGCTGGCCATGATTGCGGTGATCCTGGAACAGGCCCTGGCCCTGATCTTTGACTGGAAGCCCTTCCGGGAGACGTTCGACCG<br>CTCGGCAGTAAAGCCCATCGTCAGTTTCCTGTTCGCCGGCGGCATCGTACATGAATTGGTACGCAGTCCTCACGAAGGACATGAATTC
T17	TTAACTGGCCAGACTGAATAGTGATAGTACCTTGTTGGTTCATGGCAAACCTCGGTGGATTCAGGCAAATAGCCAGACATTGGCAAATAACGGATGACGTTGCCGGCGGCGCAGTGGGTCTGCCGGGCCAACCCCGTCCAT<br>GCTGTGACGGAGATCAAGATTGATGTCGCCGATCAGGTAATAACTGTGCTCCACCAGACCGTGTACCAGCTGACTGCAGTCGAGTTGTTGAATCTTTTGGTGCAGTGCTGCAGGCCTCGCGGCGCCGGCGGATCCGAGCCGA<br>TCCGGGTTGCCCTTGGTATAGTCTGAAATCACCAGCGCGTTGTCGCCTTTGTTGTGATACACAGTGACCTGGCGGGCCAGCTCATGAATTC
T18	TTAACTGATCGGCGACCTGGTGGGCCAGTTCGGGTGACACCCCGCGGTGCATGTAGATGTGGGCAAGCTCGCTATGTTCGGCCCTCGGGTTGCTCGCCAGCTCCGCCCGTTCCCGTGACAGGTCGGCCCGCTCGGTATCGGCT<br>TGGGAATGCACGGAAACGTATTCGCCGGCCGCCATGGACATTGCGCCCGCCACCAACCCGGCCATGCCGGTGACGAGCAGCGAAGCGTGGGTAGCGTTGGCGGCAGCGACGACGATCAGCAGGCTGGCGGTGGAGACGAT<br>ACCGTCGTTGGCCCCCAGGACTGCGGCGCGCAGCCAGCCGATACGATCACTGCGATGGGACTCGGTGTGCCGGTGCATGAATTC
T19	GAATTCATGAGCTGGCCCGCCAGGTCACTGTGTATCACAACAAAGGCGACAACGCGCTGGTGATTTCAGACTATACCAAGGGCAACCCGGATCGGCTCGGATCCGCCGGCGCCGCGAGGCCTGCAGCACTGCACCAAAAG<br>ATTCAACAACTCGACTGCAGTCAGCTGGTACACGGTCTGGTGGAGCACAGTTATTACCTGATCGGCGACATCAATCTTGATCTCCGTCACAGCATGGACGGGGTTGGCCCGGCAGACCCGCTGCGCCGCCGGCAACGTCATC<br>CGTTATTTGCCAATGTCTGGCTATTTGCCTGAATCCACCGAGGTTTGCCATGAACCAACAAGGTACTATCACTATTCAGTCTGGCCAGTTAA
T20	GAATTCAAAGAGCTGATGAAATGGTGTGTTTGTTGTATATGTGTTGATTGATCTCTCCTACGATGTTAA
T21	TTAACATATACTGAAATATTGTTGAAAATTTGATGGATGTTGAAAACTGTAATAGGCAGGATTTGAATTC

T22	TTAACTCTATGGGCTTTGGGTTATGACAAATCGTTGATATTTTATGCATATCGGCTAATGTAAGCAACTTTGAATTC
T23	GAATTCAAAGTTGCTTACATTAGCCGATATGCATAAAATATCAACGATTTGTCATAACCCAAAGCCCATAGAGTTAA
T24	AAAAAAAATCGAAAAAAAATGAAAAAAAAGAAGTGACAAAAAAAATCGAG
T25	GAATTCAGTACCGTACATTTTGGCTGTGCAATAACTCAACCACAGCAGACACTACTCCCTTTCCTTCAACAGATATATCAAATCCAGGTTCTTCAACCTTTCTGTTCAAAAGCTTGTCAAGTTCCCCACGCAGTTTCCGTATCA AGTCCAGTACACTCTTCGACGCAGAAAAATGAAGGTACCCCCCAAGCATCTCAATACCCTCTCCCGTTTTGCTAGGAATAAGATTACCACCAAACAGAAGTAAAGCGTAATCAGAAATATTTGTTGAGTCTCGGACAAAAA TGCTGGTTGTTTTGACCTTTTCACTGTAAACCATATAAGGCAATGGAAAGAGATGAACCCCCGCGTTAA
T26	GAATTCACCCGCAGTGGGTCAAAAACCCTCCGGTAGAGCTGTGGCTCAGCACTTGAGCCTGTGGTGCCCACGATGGCACCACTAGACCCCGCCCTTTTGTCCTCTCCAGAAACCCCTTTGGTAGAAAGTCGAAAGGGTCCTT CTGGCTCTCCGCACTGAAAAAAGTGGCGTTAGCAACCTTATCGTTTGGGCTTCTTACAACCCACAAGAATCGGTGCTCACTCATCTCCAACCCCACCGCCAACTCGTTTAGCTGGTTGGAAGACAGGGTCCCACCGCTCCCA AAAGACACATACAAAACCGAGCCATGTGGCTGCTTATCTAGCCACTTCAGGCAATCCGTCCCATCAGCTTTGCCTTTGTTACCCGCATCAACGTTAA
T27	GAATTCACCCGCAGTGGGTCAAAAACCCTCCGGTAGAGCTGTGGCTCAGCACTTGAGCCTGTGGTGCCCACGATGGCACCACTAGACCCCGCCCTTTTGTCCTCTCCAGAAACCCCTTTGGTAGAAAGTCGAAAGGGTCCTT CTGGCTCTCCGCACTGAAAAAAGTGGCGTTAGCAACCTTATCGTTTGGGCTTCTTACAACCCACAAGAATCGGTGCTCACTCATCTCCAACCCCACCGCCAACCCGTTTAGCTGGTTGGAAGACAGGGTCCCACCGCTCCCA AAAGACACATACAAAACCGAGCCATGTGGCTGCTTATCTAGCCACTTCAGGCAATCCGTCCCATCAGCTTTGCCTTTGTTACTCGCATCAACGTTAA
T28	GAATTCACTCATCCCAAGACAAGAAGCAAGACTGATTGTGGTGGATACTGACTCAGATAAAACCATGGTCTTGGCTCTGTCTGTACACACTGGTAGTGCAAGAAGAGACGAAGTCAAGTCTTAGTCTTTGACTTCGATTTGT ACGTGTTAA
T29	GAATTCACTCATCCCAAGACAAGAAGCAAGACTGATTGTGGTGGATACTGACTCAGATAAAACCATGGTCTTGGCTCTGTCTGTACACACTGGTAGTGCAAGAAGAGACGAAGTCAAGTCTTAGTCTTTGACTTCGATTTGT ACGTGTTAA
T30	TTAACGCCATTGCTTGTTGTTGGGAATGATGGATTGATATTGAGAACGATGTGGGTGAATTC
T31	GAATTCACCCACATCGTTCTCAATATCAATCCATCATTCCCAACAACAAGCAACGGCGTTAA
T32	TTAACGCCGTTGCTTGTTGTTGGGAATGATGGATTGATATTGAGAACGATGTAGGTGAATTC
T33	TTAACCCCACGCCCATGCAAAGTTTCTATATTTTGGCCGATTCGAAAACAGCAGGTAAATACAACATTGAATCTGTTGTCTAGGTTCTGCTAGTGAATTC
T34	TTAACCCCACACCCATGCAAAGCTTCTATATTTTGGCCGATTCGAAAACAGCAGGTAAATATAACATTGAATCTGTTGTCTAGGTTCTGCTAGTGAATTC
T35	TTAACCCCACGCCCATGCAAAGTTTCTATATTTTGGCCGATTCGAAAACAGCAGGTAAATATAACATTGAATCTGTTGTCTAGGTTCTGCTAGTGAATTC
T36	TTAACCCCACACCCATGCAAAGTTTCTATATTTTGGCCGATTCGGAAACAGCAGGTAAATACAACATTGAATCTGTTGTCTAGGTTCTGCTAGTGAATTC
T37	GAATTCACTAGCAGAACCTAGACAACAGATTCAATGTTATATTTACCTGCTGTTTTCGAATCGGCCAAAATATAGAAACTTTGCATGGGCGTGGGGTTAA
T38	GAATTCACTAGCAGAACCTAGACAACAGATTCAATGTTATATTTACCTGCTGTTTTCGAATCGGCCAAAATATAGAAACTTTGCATGGGTGTGGGGTTAA
T39	TTAACCCAATGAGCTCGCCAATTCTGGGAGGTAGCATCTTCAGCTCTGGACAAGCATACAACCTAAGTACCTGTAGTTTCTTCAGCATACCCAAATCAGCAGGCAATTCACATAGTCGGTGGCAGTTGGTTATGCTCAGGTTC TGGAGTGAATTC
T40	TTAACGGCGGCGGTGGGCGCAACGTTTTGCATGGATGACCAGAACATTTTCTGGAATTGGTCAAAGGCCTGCATGCCCATGGGCAGCCATTGTTGCATCATTTTATCGGGTTCCATTGATTGCATGCCTTGCATCATGCGTTGT TGAATTTCCTTCATCATCGCTTGTTGCATGGGTTGCACATCAGGCAGGCCGAAAAATGCGCGAGCTTCTTCGGGGGTGCATTCCATATTCACAGTGAATTC
T41	TTAACAACCGACTTTTCGGTCTCCATATTGTGGAAGCTTTGCAAGAAGGAGAAAACCGAAGATGATGCAAGTGTTATACTTGAAGCTCTTCAAGTGGGCGCTTTTCTAAAGCTTTTGCTACTTTTACAGGTTGGATGTGTTGAG GAAACTAAGGACAAAGCTTCCGAGTTGTTGAAAATATTGAATCTTCATAGGAACAAAATGGAATGTGTTGATCCAATGGATAATTTCAAGGATCTGAAAAGGCCATTTTGATTGTAAATGATACATCAGTACGTGTGAATTC
T42	GAATTCACACGTACTGATGTATCATTTACAATCAAAATGGCCTTTTCAGATCCTTGAAATTATCCATTGGATCAACACATTCCATTTTGTTTCTATGAAGATTCAATATTTTCAACAACTCGGAAGCTTTGTCCTTAGTTTCCTC AACACATCCAACCTGTAAAAGTAGCAAAAGCTTTAGAAAAGCGCCCACTTGAAGAGCTTCAAGTATAACACTTGCATCATCTTCGGTTTTCTCCTTCTTGCAAAGCTTCCACAATATGGAGACCGAAAAGTCGGTTGTTAA
T43	GAATTCAATACGTTGTGTTCTAACGCATTGGATATGACACGTTATTTTCTTGAGATGAGGATTTTTCAAAAAACAATAAAGGCAATATTTCGTATTTGAAAATTCGAGAAGTCGTGCCCTAACTTACTAGGTTTCAATTTTTCTC GCGTTAA
T44	TTAACGCAGTCATTGAATTGGTACGCAGTCACGATGAGTCCTGAGTAACAGAACCCATTTCTTGATCAAACTCAAAGCTACGTCAGGTATTGAATTC
T45	TTAACGCGACTGCTCAGAGGTATTTGAAAAAGCAATAGGATAAGTCACTTGCGGAAATATTACCTGATTCAACACATTCAGATTCGTAATTCGACGAAAGCACTCATCATTCTTTTTCGTCTTTCTTATTTACAAGTAGTGTTA TTGAATTC

T46	TTAACGCGAGAAAAATTGAAACCTAGTAAGTTAGGGCACGACTTCTCGAATTTTCAAATACGAAATATTGCCTTTATTGTTTTTTGAAAAATCCTCATCTCAAGAAAATAACGTGTCATATCCAATGCGTTAGGACACAACGTATTGAATTC
T47	GAATTCAGGGATGTAGTTTACCTGAAGTGTAGATACATGTTGTACTCGCGTTGCCAATTTTTTCTATGGATTTACTTCGGGATGCTATCGATGTAAGCTTGTTTATATGGCTGTAAATTGAATCCTAGACAGTAGTAGTTTCATGATTGTGGATGGTTAA
T48	GAATTCAGGGATGTAGTTTACCTGAAGTGTGGATACATGTTGTACTCGCGTTGCCAATTTTTTCTATGGATTTACTTCGGGATGCTATCGATGTAAGCTTGTTTATATGGCTGTAAATTGAATCCTAGACAGTAGTAGTTTCATGATTGTGGATGGTTAA
T49	GAATTCAGAAAAATACATACATATATTGAAGTTTGAATCCAAGATACTATAACATCTACCCTTCAACCTTAGATTCCAACCGAAGCTACATCTGATTTTTATGCCAGATTTCAATAAACTTATTACATCATTATTTTTACTCAGATCGTAGGTTAA
T50	GAATTCAGACGAAACAAGTTTCAATCAGACTCTAATGTCAGCAGAAACCAAAGTTCTAAGAGCATGGAAGATTTGTAGTTTTTGTTGTAAGTAATGTGCAGGACTTATTTGAGCGGCTGTAGTGATGACAAGGCCAAAGTTA
T51	TTAACATTACAGCAACGACCCGAAACATAATAACACTGAACGGCTTATCCTGGCGACGAGCTGAAAACCTCTTCAAACATCGATATGAAACCGCTTCAGAGACTGGAATCTTGCTCTTCGACCTCAAAACATCTCTTTTGCTTGTCTCGAAAATCTGGGGTTCGTCCATGGTCCTTCGAGCAGCATCGTTGCCAACACTGATATACTCTTCCATGGCTTTTACACCCTTCGTAAGGATGGGTTTCGATTCATGTTCTTTCTCTTCGAAGCGAGGTGAATGGACCACAAGTTTACACCTTCTCGATGCAATACCCTGTGTGTCGAAGTTCTCTGTGTCTGGCTGAATTC
T52	GAATTCAGCCAGACACAGAGAACTTCGACACACAGGGTATTGCATCGAGAAGGTGTAAACTTGTGGTCCATTCACCTCGCTTCGAAGAGAAAGAACATGAATCGAAACCCATCCTTACGAAGGGTGTAAAAGCCATGGAAGAGTATATCAGTGTTGGCAACGATGCTGCTCGAAGGACCATGGACGAACCCCAGATTTTCGAGACAAGCAAAAGAGATGTTTTGAGGTCGAAGAGCAAGATTCCAGTCTCTGAAGCGGTTTCATATCGATGTTTGAAGAGGTTTTCAGCTCGTCGCCAGCATAAGCCGTTCAGTGTTATTATGTTTCGGGTCGTTGCTGTAATGTTAA
T53	TTAACATGCAGAACCTCATTTTCTTCTTCCTTGGTGGAGCCATCTACAACTTGCTTTGCTTTGGGTGGATTTTCTTTCCACGGTGGTAATTCTGAAACCTTAGAATTTTCTTCTGCTGTTCCATCAATGCCAAGCTTTGCATTCTTCAGTCCTAGCTCCGTTGCAGAACAAGAGAACATTGCCCTGGTTGAGCGATTTGCTGGAGATACAGGGTTGGATAAACTGCTCGTGCTACCGGATTCTTCACTTTCCTGTGGACCTGAGGCAAGAGAAAAGGAGACCAAGACAGCTGAATTC
T54	TTAACATGCAGACCCTCATTTTCTTCTTCCTTGGTGGAGCCATCTACAACTTGCTTTGCTTTGGGTGGATTTTCTTTCCACGGTGGTAATTCTGAAACCTTAGAATTTTCTTCTGCTGTTCCATCAATGCCAAGCTTTGCATTCTTCAGTCCTAGCTCCGTTGCAGAACAAGAGAACATTGCCCTGGTTGAGCGATTTGCTGGAGATACAGGGTTGGATAAACTGCTCGTGCTACCGGATTCTTCACTTTCCTGTGGACCTGAGGCAAGAGAAAAGGAGACCAAGACAGCTGAATTC
T55	TTAACACTACAGCAACGACCCGAAACATAATAACACTGAACGGCTTATGCTGGCGACGAGCTGAAAACCTCTTCAAACATCGATATGAAACCGCTTCCGAGACTGGAATCTTGCTCTTCGACCTCAAAACATCTCTTTTGCTTGTCTCGAAAATCTGGGGTTCGTCCATGGTCCTTCGAGCAGCATCGTTGCCAACACTGATATACTCTTCCATGGCTTTTACACCCTCCGTAAGGATGGGTTTCGATTCATGTTCTTTCTCTTCGAAGCGAGGTGAATGGACCACAAGTTTACACCTTCTCGATGTAATACCCTGTGTGTCGAAGTTCTCTGTGTCTGGCTGAATTC
T56	TTAACATTACAGCAACGACCCGAAACATAATAACACTGAACGGCTTATCCTGGCGACGAGCTGAAAACCTCTTCAAACATCGATATGAAACCGCTTCAGAGACTGGAATCTTGCTCTTCGACCTCAAAACATCTCTTTTGCTTGTCTCGAAAATCTGGGGTTCGTCCATGGTCCTTCGAGCAGCATCGTTGCCAACACTGATATACTCTTCCATGGCTTTTACACCCTTCGTAAGGATGGGTTTCGATTCATGTTCTTTCTCTTCGAAGCGAGGTGAATGGACCACAAGTTTACACCTTCTCGATGCAATACCCTGTGTGTCGAAGTTCTCTGTGTCTGGCTGAATTC
T57	TTAACACGCGGTAGGGCCAAAACCGACGCTGGAAAGACCCGCCAAACCGAGCGAGACCTGCGTTGTCTGAGGCTCGTGGCAAAGAGCTTCGCCAAGGAGACGTTGAATATCGACGTCACAACGACGACCGCCTGCGTGTGCGCCATTCACGGATTTTCGGCGGTTTCCCCAGACCTCCAGGTCAGCCTGGGCGAACTGGAGGTCCGGTACGATATCGATCTGCAAAGCGTCGTCGAACGTACCTATAAGGAATTGGTACGCAGTCTACGAGACTAATTGGTACGCAGTCTACGAGTAATTGGTACGCAGTCTACGAGGCTGAATTC
T58	TTAACACCAAAACCCCATTTATCAAAAACAACAATCACAATCAAATGGGCAAAGTGGCGTACTTCCTGCACCAGATTTACTAATACCCAATTCTTCTTCAGTGTAAATTGCAAAACCATCTGCCGTTCTTTTCCGAGGCCGAGAAGAAGGTTCATTGAATCCTCCTTCCCTGTTGGTTTCTTTACTCTTTTTACTCTTCTTTTTCGACAATTTGGGTTTTGAAATTTCCTCGCCGTTCGGTTTCTCGGACCCTTTAGGGTCGGGTTTCTTCCTCTTTTTACCTGCAAATATCTCGTCGATTTCGTTACCAGCCATTTTAGAAGCTGAATTC
T59	GAATTCAGCTTCTAAAATGGCTGGTAACGAAATCGACGAGATATTTGCAGGTAAAAAGAGGAAGAAACCCGACCCTAAAGGGTCCGAGAAACCGAACGGCGAGGAAATTTCAAAACCCAAATTGTCGAAAAAGAAGAGTAAAAAGAGTAAAGAAACCAACAGGGAAGGAGGATTCAATGAACCTTCTTCTCGGCCTCGGAAAAGAACGGCAGATGGTTTTGCAATTTACACTGAAGAAGAATTGGGTATTAGTAAATCTGGTGCAGGAAGTACGCCACTTTGCCCACTTGATTGTGATTGTTGTTTTTGATAAATGGGGTTTTGGTGTTAA
T60	GAATTCAGCTTCTAAAATGGCTGGTAACGAAATCGACGAGATATTTGCAGGTAAAAAGGGGAAGAAACCCGACCCTAAAGGGTCCGAGAAACCGAACGGCGAGGAAATTTCAAAACCCAAATTGTCGAAAAAGAAGAGTAAAAAGAGTAAAGAAACCAACAGGGAAGGAGGATTCAATGAACCTTCTTCTCGGCCTCGGAAAAGAACGGCAGATGGTTTTGCAATTTACACTGAAGAAGAATTGGGTATTAGTAAATCTGGTGCAGGAAGTACGCCACTTTGCCCATTTGATTGTGATTGTTGTTTTTGATAAATGGGGTTTTGGTGTTA
T61	TTAACACCAAAACCCCATTTATCAAAAACAACAATCACAATCAAATGGGCAAAGTGGCGTACTTCCTGCACCAGATTTACTAATACCCAATTCTTCAGTGTAAATTGCAAAACCATCTGCCGTTCTTTTCCGAGGCCGAGAAGAAGGTTCATTGAATCCTCCTTCCCTGTTGGTTTCTTTACTCTTTTTACTCTTCTTTTTCGACAATTTGGGTTTTGAAATTTCCTCGCCGTTCGGTTTCTCGGACCCTTTAGGGTCGGGTTTCTTCCTCTTTTTACCTGCAAATATCTCGTCGATTTCGTTACCAGCCATTTTAGAAGCTGAATTC
T62	TTAACACCAAAACCCCATTTATCAAAAACAACAATCACAATCAAATGGGCAAAGTGGCGTACTTCCTGCACCAGACTTACTAATACCCAATTCTTCTTCAGTGTAAATTGCAAAACCATCTGCCGTTCTTTTCCGAGGCCGAGAAGAAGGTTCATTGAATCCTCCTTCCCTGTTGGTTTCTTTACTCTTTTTACTCTTCTTTCTCGACAATTTGGGTTTTGAAATTTCCTCGCCGTTCGGTTTCTCGGCCCCTTTAGGGTCGGGTTTCTTCCTCTTTTTACCTGCAAATATCTCGTCGATTTCGTTACCAGCCATTTTAGAAGCTGAATTC
T63	TTAACAGCATAGTGCCCAAAGTTCCTACCCGCAGATAGCATCCGCTGCTTATTCAATAGCTGGGTTCTTGCTTTGTTGAGGGCTGAATTC

T64	TTAACCATAGATGCCGAATGTGAGGTTGCCAGGCTAATGCTGAATTC
T65	TTAACCAAACCCTGCTACTGTTTCACTGTGTCTGTTTGTCACTTGGCTGAATTC
T66	TTAACCATGCAGACACAACAAGAAACTTTTCTGAAAGAGCGGGGTAAGTTGCTGAATTC
T67	GAATTCAGCGACATATATGAAAAAATCGAGAATAGTAAAAGGCAGCAGATGTTGGAATTGGAGAAGATGAGAATGGATTTTCACAGGGAGTTGGAAATGCAGAAGAGGCAGATCATGGAAAGAGCGCAGGCTGAAATTG CGGAACTACAGCATGGTGAGAATGAGGCAAATGATGCCTCCGGTGAGAATGCTAGTGGGTAACATATGCAAAACCCTGTTATCTTCTTTATAACATTCATCAGATTTTGGCTGTTAA
T68	GAATTCAGCCTGTTCCAAAGCAAAACGCTGAGAAGAACAACAGCAACGAGAAGGCTTAGCCATGTAGTTTGGTTGCTGAGAGTGTTATCTTATACATTGTTTTCTCCCATGCTTGCTTTCTTACTTTACTTTGCATTGGTTAA
T69	TTAACCAATGCAAAGTAAAGTAAGAAAGCAAGCACAGGAGAGAAAAATGTATAAGATAACACTCTCAGCAACCAAACTACATGGCTAAGCCTTCTCGTTGCTGTTGCTCTTCTTCTCAGCGTTTTGCTTTGGAACAGGCTGA ATTC
T70	TTAACCAATGCAAAGTAAAGTAAGGAAGCAAGCACAGGAGAAAAAAATGTATAAGATAACACTTTCAGCAACCAAACTACATGGCTAAGCTTTCTCGTTGCTGTTGCTCTTCTTCTCAGCGTTTTGCTTTGGAACAGGCTGA ATTC
T71	GAATTCAGCCTGTTCCAAAGCAAAACGCTGAGAAGAACAACAGCAACGAGAAGGCTTAGCCATGTAGTTTGGTTGCTGAGAGTGTTATCTTATACAGTGTTTTCTCCCATGCTTGCTTTCTTACTTTACTTTGCATTGGTTAA
T72	GAATTCAGCCGCCGGTTAA
T73	TTAACTCAGTAGTGTAACATACCTTACAACTCACAAGACATGTACCGCCATAACCCGAACAAGGTAAAGGTTATGCATGTCTACTTCTGAGATAATATAAGGTCTCAACCTAAACCATCTGTATAAGACAAGATTAGCTTCA ACTTGAATACGTTCATGATTCATCTCACAGCTTGCTCTTCTACTTTTCTTCCTAGCATTGAATTTGGGACACCCACGCTTGTTCTTAGATTGCAAAGCAAAGCATAACCCCCAGTCGAAACTATTTTGAAATATCCATCTACTG CCTTTGAAAGATTATCCAGCGCTTGTGATAGTTCCTCTGCGGTCCTTCTCAAATCCGATACTGAATTC
T74	GAATTCAGTGTAGTACTTGTTGTCCAGGCGATTGGGTGTGACACTGTCAAGTGGCACTGTGGTTGGATCCCCTCCAGCACCAGCCGCAGATGTTGGGGGTGGGCACTTGGTTTTCAAAAAGGCAGCGTAGTTAGGATCAAGA GAAGGGTCTTGGGCATGAGTGGCGTTAA
T75	TTAACGCAAACACCAGTGCAGCTAACTCGAGATCATGCATCGGATAATTCCTCTCATGCAACCTAAGTTGTCGGGACTCATAACCCACAACCTTACCATCTTGCATAAGTACACATCCCATCTAACATGTGATGCATCACTA TACACCACAAACTCCTTACTGAATTC
T76	TTAACGCAAACACCAGTGCAGCTAACTCGAGATCATGCATCGGATAATTCCTCTCATGCAACCTAAGTTGTCGGGACTCATAACCCACAACCTTACCATCTTGCATCAGTACACATCCCATCTAACATGTGATGCATCACTA TACACCACAAACTCCTTACTGAATTC
T77	GAATTCAGTACCGTACATTTTGGcTGTGCAATAACTCAACCACAGCAGACACTACTCCCTTTCCTTCAACAGATATATCAAATCCAGGTTCTTCAACCTTTCTGTTCAAAAGCTTGTCAAGTTCCCCACGCAGTTTCCGTATCA AGTCCAGTACACTCTTCGACGCAGAAAAATGAAGGTACCTCCCAAGCATCTCAATACCCTCTCCCGTTTTGCTAGGAGTAAGATTACCACCAAACAGAAGTAAAGCGTAATCAGAAATATTTGTTGAGTCTCGGACAAAAA TGCTGGTTGTTTTGACCTTTTCACTGTAAACCATATAAGGCAATGGAAAGAGATGAACCCCCGCGTTAA
T78	GAATTCAGTACCGTACATTTTGGCTGTGCAATAACTCAACCACAGCAGACACTACTCCCTTTCCTTCAACAGATATATCAAATCCAGGTTCTTCAACCTTTCTGTTCAAAAGCTTGTCAAGTTCCCCACGCAGTTTCCGTATCA AGTCCAGTACACTCTTCGACGCAGAAAAATGAAGGTACCCCCCAAGCATCTCAATACCCTCTCCCGTTTTGCTAGGAATAAGATTACCACCAAACAGAAGTAAAGCGTAATCAGAAATATTTGTTGAGTCTCGGACAAAAA TGCTGGTTGTTTTGACCTTTTCACTGTAAACCATATAAGGCAATGGAAGGAGATGAACCCCCGCGTTAA
T79	TTAACGTCAGTGAGAAGAACTTTGGCGCCAAGTTCTATCAGAAGCCTAGATGTTGCAGCGCCAATGCCGCTTGCGGCGCCAGTGACGACAGCGACTTTTTGCGACCAATCCATCAGCTGACTCACATCGCTTCAACTCAAAA TGCGAAGAGCCGTCGCTAGCGGCGACGGCTCTCCCGGCTAAATTAGAACTGATAGCTAAAATTAGCGCCGATTGTTCGAGGCTCACCCGTAAATCGAACAGCACCATCGCCCGCAGAAGCTACTGAATTC
T80	GAATTCAGTGTGCACAACCCGCAACAAGGGGTGAATGGCGCAACATCGGTGCGACAACCGTGACAGTTCCGAATGCGGGCCCAGGTCGGTGCACGCCGCGTGTCACGCCTCGTGTACTACGTCTGGGACGACGGGTGGGC GGCATCATTGCCTGCCAGTTGCTGTTGACGCGAAGATCGACCTCACGGATGGTCGCTCACAGCGCTGCACTCGCACCACCCTCGGTGTGCATGCGTGTTCGGAATGCACGTTAA
T81	TTAACGTCAGTGAGAAGAACTTTGGCGCCAAGTTCTATCAGAAGCCTAGATGTTGCAGCGCCAATGCCGCTTGCGGCGCCAGTGACGACAGCGACTTTTTGCGACCAATCCATCAGCTGACTCACATCGCCTCCACTCAAAA TGCAAAGAGCCGTCGCTAGCGGCGACGGCTCTCCCGGCTAAATTAGAACTGATAGCTAAAATTAGCGCCGATTGTTCGAGGCTCACCCGTAAATCGAACAGCACCATCGCCCGCAGAAGCTACTGAATTC
T82	GAATTCAGTGTGCACAACCCGCAACAAGGGGTGAATGGCGCAACATCGGTGTGACAACCGTGGCAGTTCCGAATGCGGGCCCAGGTCGGTGCACGCCGCGTGTCACGCCTCGTGTACTACGTCTGGGACGACGGGTGGGCG GCATCATTGCCTGCCAGTTGCTGTTGACGCGAAGATCGACCTCACGGATGGTCGCTCACAGCGCTGCACTCGCACCACCCTCGGTGTGCATGTGTGTTCGGAATGCACGTTAA
T83	TTAACGTGCATTCCGAACACACATGCACACCGAGGGTGGTGCGAGTGCAGCGCTGTGAGCGACCATCCGTGAGGTCGATCTTCGCGTCAACAGCAACTGGCAGGCAATGATGCCGCCCACCCGTCGTCCCAGACGTAGTAC ACGAGGCGTGACACGCGGCGTGCACCGACCTGGGCCCGCATTCGGAACTGTCACGGTTGTCACACCGATGTTGCGCCATTCACCCCTTGTTGCGGGTTGTGCACACTGAATTC
T84	GAATTCAGTGTGCACAACCCGCAACAAGGGGTGAATGGCGCAACATCGGTGTGACAACCGTGACAGTCCCGAATGCGGGCCCAGGTCGGTGCACGCCGCGTGCCACGCCTCGTGTACTACGTCTGGGACGACGGGTGGGC GGCATCATTGCCTGCCAGTTGCTGTTGACGCGAAGATCGACCTCACGGATGGTCGCTCACAGCGCTGCACTCGCACCACCCTCGGTGTGCATGTGTGTTCGGAATGCACGTTAA
T85	GAATTCAGTCATAGAGATCCGAAGTTTTCTGGGGTTGGCAGGTTATTATCGGAGATTCGTAGAAGGATTTTCTGTGTTGGCAGCAACGTTAA
T86	GAATTCAGTGTAGTAAGAAAATGATCGGTTTTTTTATTTTTATTTTTTTATGTGCGAGATAAATTGTGGCAATGAGTTTTTTCCACGTTAA
T87	GAATTCAGTAAGATATGACAGAAAAGAAGCGGGAGTCTCAGAATGACATGATGGCACGTTAA
T88	TTAACGTCGTTAGCTCAACGCTGTGAATGGTTATATGGTGGCTCTAACTGAATTGGTACGCAGTCATGATGAGTCCTGAGTAACGTGTTTCCAATGAATTGAAAGCCAAGAACGAATAGAAACCCAACTTACCACCAACGAT AACCCTTCGTTTAACTATTGTATATACGAGAACACTGAATTC
T89	TTAACCATTCCGTGACTTTGGCACTGGACGTTCCAAAAATGGGTACTATCGGGTCGGGTGAATTC

T90	GAATTCACCGATTCTCCCATCAAACATGTATGCCAGTGCTAATGCAGCCATGAAGCGTGCCATCTTGGATAACGATGAAGAACAAAGATGAACGAGTGCAGGAACCCCACCTTCTTCCACTATCCGAAGTGCATTACTATG<br>GTTACTCAGGACTCATCATGACTGCGTACCAATTCATCAAATCTAGTGGACATGGCGCTGTTTTCATGGATGGTTCATAGTTCTAGCAACATCAGCTCAGTAAGGAAGAAAGAAATCTATCAGTATATTTGGTTACTCAGGAC<br>TCATCGTGACTGCGTACCAATTCACCACATTGTTCAAGAACAAACAAATCAACAACTTCAATGTTCTAGCCAAACATATATATTGACAAAGAAACAGATATCCATCAAGTATCGAGATAATTATGCCGAGAAATCAATTTTG<br>TCACTATTGATGTCTTTGATGGAGATGGAGGTTTATATGGTGGTTAA
T91	GAATTCACCATGATTTTGTATTAGAGAAACATACAGCATTCCTATGTTTCCATAATTGCCAAACGGTGGATATCCAGCCTTCGATTGTTGATTTGCCAATGGTTAA
T92	GAATTCACCAAAAAGACTCAAAAATCCATAATTCGTTGTCTTCCCGTAATGGATTTTAGTCAGCCTCCAATTGAAAATTATGCCAACCCCAAGACTTGTTTCTTTCATGTTCTCTTCAAGGCTGCAGCATTGGCTTTTTACATC<br>CTTTCAGCTCTCTTTTTCAACAGCTTTGTGATCATATTTGTGGTGACTGTTCTTCTTTCCGCCCTTGATTTTTGGGTGGTTAA
T93	TTAACCAGTCCATGCCCAAGATAATATCAAATTCCCGGAAGGGTAATAACATCAAATCAGTGGTGAATTC
T94	TTAACCAAATAGTTGTAGCCTCAAAAGACCAACAGAAAATGAATTTTACTTGCCCGTACGGTACGTTTGCTTATAGGTGAATTC
T95	TTAACTCAATTGCTGCCATTATTATGCATTGAAGATAAGCATATTGGAATTTGATTATAAAAAGGAAAAGAAGCAATAGCACTTTATATTTGTAACTTCTGTTGCTGCAAAATAGCTTTCAATCAGTTCTTCTTTGGAACCCCG<br>AGCCTCAGTCGGAAATCCTCCTCCATAAGTGGAAGTCCGTCACGCAACTTCACCTTTGGTTCCCATCCGAGCACCTCTTTTGCTTTGGTTATGTCCGGCTTCCTTTGTCGGGGATCATCAGGAGTGTTTTCCACCATCGATATCT<br>TCACCTCAGGATTGATAAGCTCCTTCACGGATTCTGCAAGCTCGAGCATGGTGAATTC
T96	GAATTCACCATGCTCGAGCTTGCAGAATCCGTGAAGGAGCTTATCGATCCTGAGGTGAAGATATCGTTGGTGGAAAACACTCCCGATGATCCCCGACAAAGGAAGCCGGACATAACCAAAGCAAAAGAGGTGCTCGGATG<br>GGAGCCAAAGGTGAAGTTGCGTGACGGGCTTCCGCTTATGGAGGAGGATTTCCGTCAGAGGCTCGGGGTCCCAAAGAAGAACTGATTGAAAGTTATTTTACAGCAACAGAAGTTACAAATATAAAGTACTATTGCTTCTTTT<br>CCTTTCTATAATCAAATTCCAATATGTTTATCTTCAATGCATAATAATGGCAGCAATGGAGTTAA
T97	GAATTCACCATGCTCGAGCTTGCAGAATCCGTGAAGGAGCTTATCGATCCTGAGGTGAAGATATCGTTGGTGGAAAACACTCCCGATGATCCCCGACAAAGGAAGCCGGACATAACCAAAGCAAAAGAGGTGCTCGGATG<br>GGAGCCAAAGGTGAAGTTGCGTGACGGGCTTCCGCTTATGGAGGAGGATTTCCGTCAGAGGCTCGGGGTCCCAAAGAAGAACTGATTGAAAGTTATTTTACAGCAACAGAAGTTACAAATATAAAGTACTATTGCTTCTTTT<br>CCTTTCTATAATCAAATTCCAATATGTTTATCTTCAATGCATAATAATGGCAGCAATGGAGTTAA
T98	GAATTCACCAGCTGAGAGAAAGATCGAATAAAGAGTGACATAAATCCGCTTTGGTTTCAAGTCAGGAATAAATAAGCAGTAGAGTTAA
T99	GAATTCACCCCCTCTTGGCGTTTTCGGTTTGTTCTATCGAGTTAA
