# Supplementary material for: Transcriptomic and proteomic analyses of a new cytoplasmic male sterile line with a wild Gossypium bickii genetic background
Source: BMC Genomics. 2020 Dec 2;21:859. doi: 10.1186/s12864-020-07261-y (PMC7709281; doi:10.1186/s12864-020-07261-y)
Supplement: Supplementary file 9 — Additional file 9: Table S3. Bud developmental stages in cotton [16]. [file 12864_2020_7261_MOESM9_ESM.pdf]

Table S3. Bud developmental stages in cotton [16].

No.	Stage	Bud transverse diameter (BTD)(mm)
1	Sporogonium stage	BTD≤1.50
2	Sporogenous cells stage	1.50＜BTD≤2.16
3	Microsporocyte stage	2.16＜BTD≤2.60
4	Meiosis stage	2.60＜BTD≤4.60
5	Tetrad stage	4.60＜BTD≤5.90
6	mononuclear and binucleate pollens stage	5.90＜BTD≤9.93
7	Pollen maturation stage	BTD＞9.93
