# Supplementary material for: Transcriptomic and proteomic analyses of a new cytoplasmic male sterile line with a wild Gossypium bickii genetic background
Source: BMC Genomics. 2020 Dec 2;21:859. doi: 10.1186/s12864-020-07261-y (PMC7709281; doi:10.1186/s12864-020-07261-y)
Supplement: Supplementary file 10 — Additional file 10: Table S4. Primers used in qRT-PCR. [file 12864_2020_7261_MOESM10_ESM.pdf]

Table S4. Primers used in qRT-PCR.

Primer number	Primer sequences	Primer number	Primer sequences
0013-F	5′ - GGATGGCGAAGCTTTTGGTC-3′	22-14R	5'-CTCACCTTCAGCCACCGATT-3'
0013-R	5′ - ATATCCACCAATCGCGAGCC 3′	15-9F	5'-GCCAATTCTGGGAGGTAGCA-3'
1003-F	5′ - GCTCCTCAGGCAGTCAAGAG-3′	15-9R	5'-CCAACTGCCACCGACTATGT-3'
1003-R	5′ - AAGCTTGTCCCCCAAAGGTC-3′	B-25F	5'-ACCCACTAGCATTCTCACCG-3'
1604-F	5′ - CGACCTTGCCTCTGCTACAA-3′	B-25R	5'-ACCCACTAGCATTCTCACCG-3'
1604-R	5′ - TAACGATTCGAGGTTGGGGC-3′	B-65F	5'-TGTCAAGTGGCACTGTGGTT-3'
2005-F	5′ - TGCGTTGCCTGAGAATGCTA-3′	B-65R	5'-CACTCATGCCCAAGACCCTT-3'
2005-R	5′ - GGTGGCAGTTTCTCCACAGT-3′	B-66F	5'-TAACGCAAACACCAGTGCAG-3'
3004-F	5′ - TCCAAACTTCCCCCAAGAGC-3′	B-66R	5'-GTGGGTTATGAGTCCCGACA-3'
3004-R	5′ -TTCCTTGCGGAGATGAGCAG-3′	B-79F	5'-GACAACCGTGACAGTTCCGA-3'
4702-F	5′ -ATGCCTGCTTTGACCGAGAT-3′	B-79R	5'-GAGGTCGATCTTCGCGTCAA-3'
4702-R	5′ -GCAAGGTCACGTCCCTCATT-3′	14-20F	5'-ATGGGACGGATTGCCTGAAG-3'
4713-F	5′ - TGCCGCTCAGTTGAAAGCTA-3′	14-20R	5'-CACTCATCTCCAACCCCACC-3'
4713-R	5′ -TGGCAGTGGTGAATATTGTGGT-3′	NC1	5'-GCGATCTGGTAAGGAGCTTG-3′
22-14F	5'-TGAAGTAGTGACAGCAGCGG-3'	NC2	5′ -GGAGAAGGTTTCCACAACCA-3′
